# Supplementation of calcium, magnesium, phosphate, and potassium in critically ill patients: A multicenter cohort study

**DOI:** 10.1371/journal.pone.0349145

**Published:** 2026-07-02

**Authors:** Christopher J. Yarnell, Thomas Bodley, Anica C. Law, George Tomlinson, Emily A. Vail, Nicholas A. Bosch

**Affiliations:** 1 Department of Critical Care Medicine, Scarborough Health Network, Toronto, Ontario, Canada; 2 Scarborough Health Network Research Institute, Toronto, Ontario, Canada; 3 Interdepartmental Division of Critical Care Medicine, University of Toronto, Toronto, Ontario, Canada; 4 The Pulmonary Center, Boston University Chobanian & Avedisian School of Medicine, Boston, Massachusetts, United States of America; 5 Department of Medicine, University Health Network, Toronto, Ontario, Canada; 6 Department of Anesthesiology and Critical Care, University of Pennsylvania Perelman School of Medicine, Philadelphia, Pennsylvania, United States of America; 7 Evans Center For Implementation and Improvement Sciences, Department of Medicine, Boston University Chobanian & Avedisian School of Medicine, Boston, Massachusetts, United States of America; The University of Texas Rio Grande Valley, UNITED STATES OF AMERICA

## Abstract

**Objective:**

To investigate hospital-level variation in the supplementation of calcium, magnesium, phosphate, and potassium in critically ill patients.

**Design:**

Retrospective cohort study.

**Setting:**

Premier Healthcare Database of inpatients from the USA.

**Patients:**

Adults (age ≥ 18 years) admitted to an intensive care unit (ICU) on hospital day 1 between October 1, 2022 and July 31, 2024, who had at least one calcium (total or ionized), magnesium, phosphate, or potassium level measured in an ICU between day 2 and 28. We excluded patients with renal failure, a pregnancy-related diagnosis, and intracranial bleeding on admission.

**Interventions:**

Calcium, magnesium, phosphate, or potassium supplementation.

**Measurements and main results:**

We included 47,988 patients from 67 ICUs across 52 hospitals, totaling 167,621 patient-days with a measurement of one of the studied electrolytes. Median age was 64 years (interquartile range [IQR] 52–74) and 46% (22,057) were female. There were 38,621 patients (80.5%) with a minimum value below a lower limit of normal, and 33,626 (70.1%) patients who received supplementation. Across hospitals, the median (IQR) percentages receiving supplementation per day were: calcium 9.4% (6.7–12.2); magnesium 28.6% (22.6–35.0); phosphate 19.1% (15.9–23.2); potassium 28.9% (25.8–34.6). From a multilevel model, the median relative odds of supplementation, comparing any two hospitals, ranged from 1.51 (95% confidence interval [CI] 1.48–1.55) for potassium to 2.09 (95% CI 1.99–2.19) for magnesium. At most hospitals, there were no calcium or phosphate levels at which the percentage receiving supplementation exceeded 50%. At 75% of hospitals there was a magnesium level at which the percentage receiving magnesium supplementation was 50% or more (range: 1.5–2.1 mg/dL), and at 96% of hospitals there was an analogous potassium level (range: 3.3–3.9 mmol/L).

**Conclusion:**

Supplementation of calcium, magnesium, phosphate, or potassium is common in critically ill patients and varies across hospitals, suggesting a need for randomized trials to clarify optimal supplementation practices.

## Introduction

Calcium, magnesium, phosphate, and potassium play important roles in maintaining homeostasis in critically ill patients, and yet low serum levels are common [1–3]. While each electrolyte has unique roles and physiology, all four are essential to neurologic function, cardiac contraction and rhythm, and energy metabolism [4–7]. Deficiencies may influence survival and recovery in critical illness [3,8–14].

Consequently, measurement and supplementation of calcium, magnesium, phosphate, and potassium levels is a routine element of supportive care in critically ill patients [15–17]. In many intensive care units (ICUs), supplemental doses of these electrolytes are administered in response to measured levels in a protocolized fashion [1,2,17–19]. However, serum levels or supplementation practices that best support recovery are unknown [20,21].

Uncertainty in optimal electrolyte supplementation practices could lead to variation across hospitals, which would signal an opportunity to better determine best practice. Substantial variation could imply opportunities for reducing unnecessary supplementation or increasing beneficial supplementation, and help define equipoise for evaluation of electrolyte supplementation targets in randomized controlled trials. Alternatively, minimal variation may suggest that clinicians have arrived at an implicit consensus regarding the optimal approach. To investigate hospital-level variation in measurement and supplementation of calcium, magnesium, phosphate, and potassium in critically ill patients, we performed a retrospective analysis of patients from a large and representative American database.

## Methods

### Design and data source

We performed a retrospective cohort study using the Premier Healthcare Database, which captures approximately 25% of United States hospitalizations. The Premier Healthcare Database makes deidentified patient-level clinical and cost data available to independent investigators for a fee. The data source for this study was the 15% of hospitals in the database who contribute time-stamped laboratory value and electrolyte administration data [22]. This study was deemed not human subjects research by the Boston University Institutional Review Board (#H-43331). Data were accessed August 7, 2025, and authors did not have access to information that could identify individual participants in this deidentified database.

We focused on levels and supplementation of calcium, magnesium, phosphate, and potassium, because these electrolytes are commonly measured in critically ill patients and commonly included in serum level-based supplementation protocols ordered at ICU admission [2,15,18]. We did not include other electrolytes such as sodium, chloride, bicarbonate, or zinc, which are uncommonly supplemented in a protocolized fashion.

### Patients

We included adult patients (age ≥ 18 years) who were admitted to a participating ICU on hospital day 1 (between October 1, 2022 and July 31, 2024) and had at least one calcium, magnesium, phosphate, or potassium level measured in an ICU between day 2 and 28 of hospitalization. We excluded patients with diagnoses of acute or chronic renal dysfunction [23,24] (Sect 2 in S1 Appendix) because electrolyte supplementation may be less common in such patients; patients with a pregnancy-related diagnosis (ICD-10 O00-O9A) because of magnesium administration for pre-eclampsia [25]; and patients with intracranial bleeding because some centers may administer magnesium to aid in cerebral hemostasis [26,27]. Because the Premier Healthcare Database does not link encounters from multiple hospitals for a single patient, we considered each admission as a separate patient in analyses.

To reduce noise in analyses of hospital-level variation, we excluded patients admitted to hospitals with fewer than 1,000 measurements of any of the studied electrolytes, and excluded hospitals for a given electrolyte if there were fewer than 50 measurements of that electrolyte from patients at that hospital [2]. We followed each patient from the day after ICU admission (day 2) until the earliest of death, ICU discharge, or day 28. We excluded the first day of ICU admission because electrolyte supplementation during acute resuscitation may differ from supplementation during stabilization and recovery [2].

### Describing serum electrolyte levels

For serum levels of total calcium, ionized calcium, magnesium, phosphate, and potassium, we collected the daily minimum value for each patient. Measurements above the upper limit of normal and below the 0.1^th^ percentile were excluded. We summarized the distribution of daily minimum electrolyte levels using mean, median, quantiles, and histograms. We compared values to the lower limits of normal established by the American Board of Internal Medicine (ABIM) (eTable 1 in S1 Appendix) [28]. Using multilevel linear regression with clustering by patient and hospital, we calculated the intraclass correlation coefficient at the hospital level to estimate the proportion of total variance attributable to individual-level as opposed to hospital-level variation. We did not attempt to convert between total and ionized calcium levels [29].

### Reporting electrolyte supplementation practices

We summarized the number and percentage of patient-days where calcium, magnesium, phosphate, and potassium supplementation were given. For the denominator of the percentage, we included all patient-days where the corresponding electrolyte was measured. For each electrolyte, we reported the hospital-level distribution of the highest serum levels at which the supplementation percentage was 50% or more. We also reported the costs of supplementation in total, per hospital, and per patient.

Using multilevel logistic regression, we modeled the association between serum level and supplementation on each patient-day, with clustering by hospital in the form of both random intercept (which allows each hospital to have a different baseline probability of supplementation) and random slope (which allows each hospital to have a different decrease in the supplementation probability as levels increase).

With the outputs of the multilevel regression, we calculated the median odds ratio to quantify variability across hospitals [30]. For a patient with a given serum level, this is the median ratio of the odds of supplementation at a hospital with a higher supplementation use to the odds at a hospital with lower supplementation use [31,32]. Each median odds ratio was calculated at the corresponding mean observed serum level.

All analyses were performed using R (version 4.0.5.) [33]. We reported uncertainty using interquartile ranges (IQRs) and 95% confidence intervals (CI).

## Results

We included 47,988 patients from 67 ICUs across 52 hospitals, totaling 167,621 patient-days with a measured serum calcium (total or ionized), magnesium, phosphate, or potassium (eFig 1 in S1 Appendix). Median age was 64 years (IQR 52–74) and 46% (22,057) were female (Table 1). Respiratory dysfunction (22.6%) and sepsis (21.4%) were commonly present on admission. A total of 15,992 (33.2%) had major procedures or surgeries on the day of admission (eTable 2 in S1 Appendix). Most patients were cared for in the East North Central or South Atlantic census divisions, in hospitals with at least 500 beds (eTable 3 in S1 Appendix).

**Table 1:** Patient characteristics.

Characteristic	Total (n = 47,988)	Hypocalcemia – total calcium (n = 32,925)	Hypocalcemia – ionized calcium (n = 7,765)	Hypomagnesemia (n = 5,110)	Hypophosphatemia (n = 19,201)	Hypokalemia (n = 15,213)
Age, years – median (IQR)	64 (52, 74)	64 (52, 73)	64 (52, 72)	62 (48, 72)	63 (50, 76)	63 (50, 73)
Sex, No. (%)						
Female	22,057 (46.0)	14,765 (44.8)	3,223 (41.5)	2,617 (51.2)	8,846 (46.1)	7,894 (51.9)
Male or unknown	25,933 (54.0)	18,160 (55.2)	4,542 (58.5)	4,293 (48.8)	10,355 (53.9)	7,319 (48.1)
Organ dysfunction present on admission, No. (%)						
Cardiovascular	8,630 (18.0)	7,133 (21.7)	1836 (23.6)	1481 (29.0)	4770 (24.8)	3875 (25.5)
Respiratory	10,843 (22.6)	8,080 (24.5)	2022 (26.0)	1472 (28.8)	5743 (29.9)	4839 (31.8)
Neurologic	6,274 (13.1)	4,642 (14.1)	1004 (12.9)	927 (18.1)	3578 (18.6)	3110 (20.4)
Hematologic	3,366 (7.0)	2,816 (8.6)	820 (10.6)	634 (12.4)	1769 (9.2)	1433 (9.4)
Hepatic	502 (1.0)	471 (1.4)	189 (2.4)	115 (2.3)	335 (1.7)	315 (2.1)
Sepsis present on admission, No. (%)	10,265 (21.4)	7,915 (24.0)	1744 (22.5)	1603 (31.4)	5566 (29.0)	4812 (31.6)
Mechanical ventilation on admission, No. (%)	16,183 (33.7)	12,708 (38.6)	3,645 (46.9)	1,820 (35.6)	7,778 (40.5)	6,141 (40.4)
Major Procedure or Surgery on admission, No. (%)	15,992 (33.3)	12,006 (36.5)	3,303 (42.5)	1,374 (26.9)	5,794 (30.2)	3,565 (23.4)
Comorbidities present on admission, No. (%)						
Alcohol use disorder	5,523 (11.5)	4307 (13.1)	1032 (13.3)	1103 (21.6)	2880 (15.0)	2370 (15.6)
Anemia	7,383 (15.4)	5416 (16.4)	1062 (13.7)	1057 (20.7)	3408 (17.7)	3061 (20.1)
Cardiac arrhythmia	15,799 (32.9)	10742 (32.6)	2570 (33.1)	1549 (30.3)	6156 (32.1)	5066 (33.3)
Chronic pulmonary disease	16,141 (33.6)	10718 (32.6)	2585 (33.3)	1670 (32.7)	6388 (33.3)	5001 (32.9)
Coagulopathy	4,923 (10.3)	4024 (12.2)	1170 (15.1)	838 (16.4)	2447 (12.7)	1975 (13.0)
Complicated diabetes	7,258 (15.1)	5004 (15.2)	1273 (16.4)	1004 (19.6)	3007 (15.7)	2520 (16.6)
Congestive heart failure	13,764 (28.7)	9,252 (28.1)	2,469 (31.8)	1,264 (24.7)	5,041 (26.3)	4,713 (31.0)
Dementia	2,484 (5.2)	1716 (5.2)	291 (3.7)	293 (5.7)	1142 (5.9)	1019 (6.7)
Fluid and electrolyte disorders	17,476 (36.4)	13187 (40.1)	2916 (37.6)	2697 (52.8)	8917 (46.4)	8066 (53.0)
Hemiplegia	3,200 (6.7)	1966 (6.0)	331 (4.3)	312 (6.1)	1306 (6.8)	1126 (7.4)
HIV/AIDS	131 (0.3)	92 (0.3)	15 (0.2)	21 (0.4)	58 (0.3)	46 (0.3)
Hypertension	33,623 (70.1)	22462 (68.2)	5478 (70.5)	3375 (66.0)	12616 (65.7)	10414 (68.5)
Liver disease	4,213 (8.8)	3464 (10.5)	835 (10.8)	851 (16.7)	2185 (11.4)	1817 (11.9)
Metastatic cancer	2,219 (4.6)	1603 (4.9)	365 (4.7)	325 (6.4)	1076 (5.6)	724 (4.8)
Peripheral vascular disease	6,579 (13.7)	4905 (14.9)	1489 (19.2)	628 (12.3)	2536 (13.2)	2025 (13.3)
Pulmonary circulation disorder	4,507 (9.4)	3061 (9.3)	807 (10.4)	445 (8.7)	1688 (8.8)	1545 (10.2)
Tumor	5,366 (11.2)	3907 (11.9)	873 (11.2)	697 (13.6)	2460 (12.8)	1641 (10.8)
Teaching hospital, No. (%)	38,805 (80.9)	26616 (80.8)	6855 (88.3)	4148 (81.2)	15595 (81.2)	11949 (78.5)
Urban hospital, No. (%)	46,414 (96.7)	31867 (96.8)	7241 (93.3)	4931 (96.5)	18335 (95.5)	14713 (96.7)
Hospital bed count, No. (%)						
100-199	1,436 (3.0)	997 (3.0)	71 (0.9)	150 (2.9)	647 (3.4)	449 (3.0)
200-299	2,797 (5.8)	1849 (5.6)	156 (2.0)	261 (5.1)	1091 (5.7)	1012 (6.7)
300-399	6,777 (14.1)	4620 (14.0)	541 (7.0)	774 (15.1)	2220 (11.6)	2341 (15.4)
400-499	10,909 (22.7)	6857 (20.8)	1416 (18.2)	910 (17.8)	3518 (18.3)	3441 (22.6)
500+	26,069 (54.3)	18602 (56.5)	5581 (71.9)	3015 (59.0)	11725 (61.1)	7970 (52.4)

Legend: This table shows the number (percentage %) of patients in the study with each characteristic. Major procedure or surgery on admission is based on Healthcare Cost and Utilization Project (HCUP) codes. Cardiac arrhythmia and fluid and electrolyte disorders were identified based on ICD codes (Sect 2 in S1 Appendix). Region of hospital available in eTable 2. IQR = interquartile range, HIV/AIDS = human immunodeficiency virus/acquired immunodeficiency syndrome.

### Electrolyte levels

Across the 167,621 patient-days with electrolyte measurements, potassium was the most commonly measured electrolyte (164,279 patient-days), and ionized calcium was the least commonly measured (27,495 patient-days) (Table 2). The number of patients who had each electrolyte value measured at least once ranged from 12,310 (25.7%, ionized calcium) to 47,611 (99.2%, potassium).

**Table 2:** Electrolyte levels and supplementation practices.

Electrolyte (patient-days)	Serum electrolyte measurement distribution					Electrolyte supplementation characteristics	
	Median	IQR	Range	Below normal range – count (%)	Patient/Hospital intraclass correlation coefficient^1^ - %	Supplementation – No. (%)	Supplementation costs,^2^ median (IQR) - $
**Calcium (n = 162,540) – mg/dL**	8.4	8.0-8.8	5.4-10.2	96,608 (59.4)	55.6 / 3.8	17,880 (10.9)^3^	21.8 (7.8, 49.5)
**Ionized calcium (n = 27,495) – mmol/L**	1.10	1.05-1.15	0.24-1.23	15,585 (56.7)	27.0 / 7.2		
**Magnesium (n = 122,921) – mg/dL**	2.0	1.8-2.2	0.8-2.6	6,571 (5.3)	34.9 / 7.2	38,092 (31.0)	13.2 (6.0, 24.4)
**Phosphate (n = 92,050) – mg/dL**	3.0	2.4-3.5	0.7-4.5	45,160 (49.1)	28.9 / 1.5	18,128 (19.7)	12.7 (3.0, 28.5)
**Potassium (n = 164,279) – mEq/L**	3.9	3.6-4.2	2.3-5.0	29,103 (17.7)	32.8 / 2.2	50,337 (30.6)	6.6 (1.95, 17.2)

Legend: This table shows information about calcium (total and ionized), magnesium, phosphate, and potassium levels and supplementation practices. Recall that the study does not include electrolyte values greater than the upper limit of normal, which means distributions may be skewed towards lower electrolyte level values. ICU = intensive care unit, IQR = interquartile range. To convert units to mmol/L, divide by 4.01 (total calcium), 2.44 (magnesium), 3.1 (phosphate).

^1^Intraclass correlation coefficients for clustering within the same patient (first number), and for clustering of patients within the same hospital (second number).

^2^Per patient-day of supplementation.

^3^Among patient-days with calcium or ionized calcium measured (n = 163,939; 26,096 patient-days had both measured).

**Fig 1:**
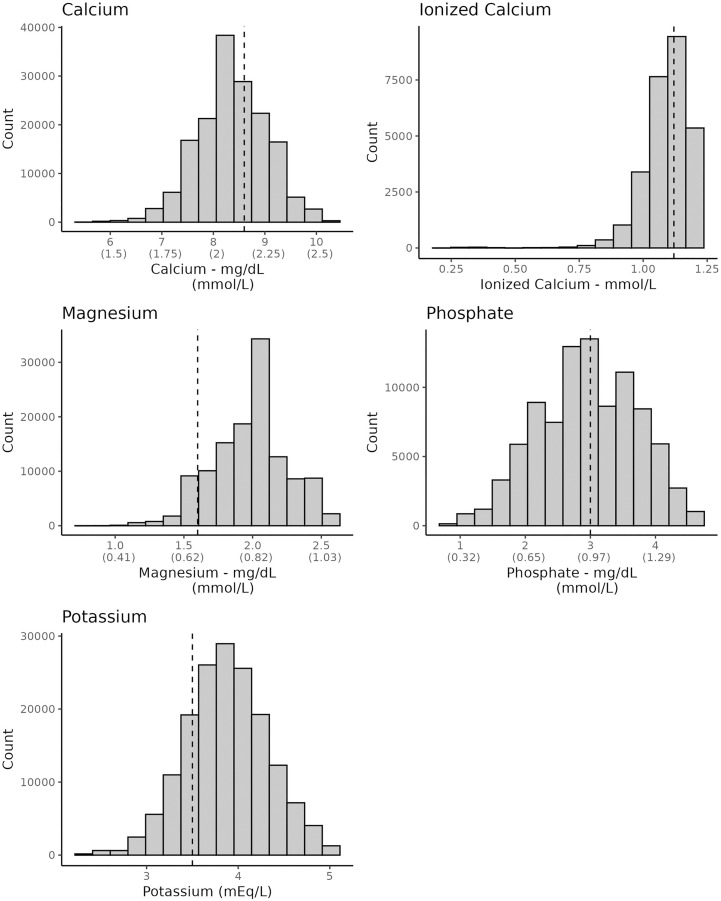
Serum levels of calcium (total and ionized), magnesium, phosphate, and potassium. This 5-panel figure shows histograms of the number of measurements (y-axis), for each interval of serum level (x-axis), for each electrolyte. For calcium, magnesium, and phosphate, the SI units are written below in parentheses. Dashed lines show the lower limit of normal, according to the American Board of Internal Medicine reference ranges.

For 38,621 patients (80.5%), there was at least one patient-day where the minimum level for one of the electrolytes fell below the lower limit of normal. The median value across patient-days was within the normal range for magnesium and potassium, but at or below the lower limit of normal for phosphate and calcium (Fig 1). The percentage of patient days with a level below the lower limit of normal ranged from 5.3% (magnesium) to 59.4% (total calcium). On almost half of all patient-days with a measured phosphate level, the level was less than 3 mg/dL. On more than half of all patient-days with a measured ionized or total calcium level, the level fell below the lower limit of normal. Electrolyte levels were stable over the course of ICU admission (eFig 2 in S1 Appendix).

### Variation in electrolyte levels across hospitals

For all four electrolytes, variation in serum levels between individuals was greater than variation in serum levels between hospitals (eFig 3 in S1 Appendix). Intraclass correlation coefficients between two measurements within the same hospital were 3.8% (total calcium), 7.2% (ionized calcium), 7.2% (magnesium), 1.5% (phosphate), and 2.2% (potassium).

### Supplementation practices

Across the 167,621 patient-days, 124,437 electrolyte supplementations were administered to 33,626 (70.1%) patients. The per patient-day percentage receiving supplementation varied from 10.9% (calcium) to 31.0% (magnesium). The most common supplementation route was parenteral for calcium, magnesium, and phosphate, and oral for potassium (eTable 4 in S1 Appendix contains additional details about administered supplements). Supplementation percentages were generally similar across time during ICU admission (eFig 4 in S1 Appendix).

**Fig 2:**
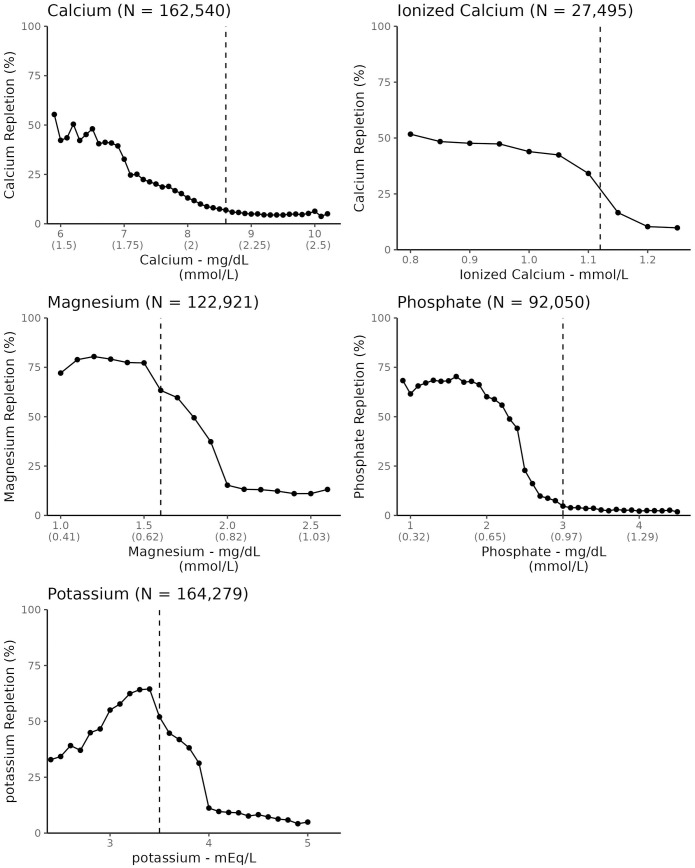
Probability of supplementation according to serum level. This five-panel figure shows the percentages (y-axis) of patients receiving a dose of each electrolyte according to the lowest serum level measured on that day (x-axis). Each header shows the number of measurements (N) included in that plot. For calcium, magnesium, and phosphate, the SI units are written below in parentheses.

Although lower serum electrolyte levels generally corresponded to higher supplementation use (Fig 2), the maximal rates of supplementation and the shapes of the curves relating supplementation to serum levels varied by electrolyte. For calcium (total and ionized), peak supplementation use was approximately 50%, even at levels below the lower limit of normal, and the decrease in supplementation use with increasing calcium levels appeared linear. For magnesium and phosphate, there was a non-linear decrease in supplementation use in the region of the lower limit of normal (magnesium) or just below the lower limit of normal (phosphate). For potassium, the supplementation percentage peaked at 3.4 mmol/L, and was negligible for all potassium levels greater than 4 mmol/L.

### Variation in supplementation across hospitals

**Fig 3:**
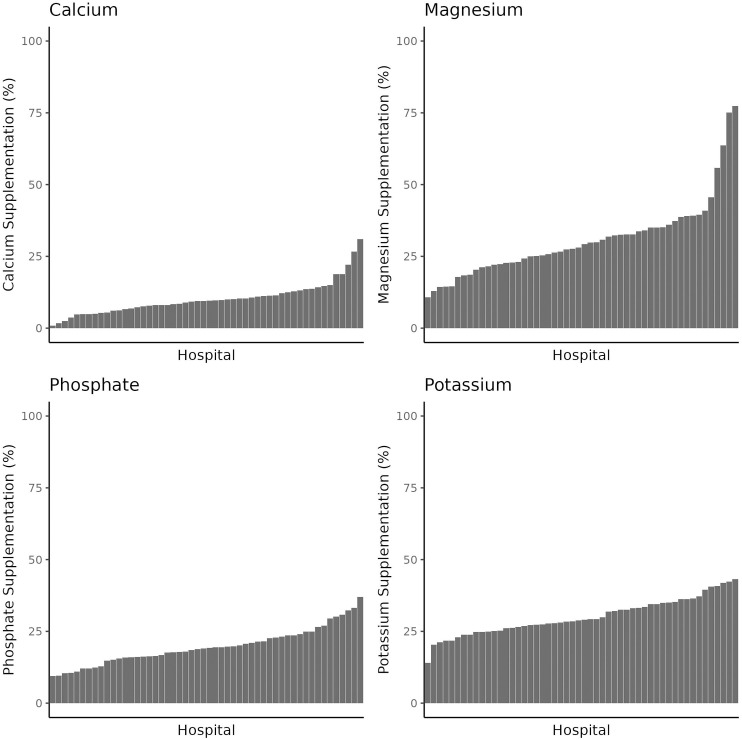
Supplementation use by hospital. This figure shows the overall percentage use of supplementation among patient-days where the corresponding electrolyte level was measured. Note that either ionized or total calcium levels were considered a patient-day in the denominator for the “Calcium” panel. Across the four electrolytes, the medians (interquartile ranges) across hospitals of percentages receiving supplementation were: calcium 9.4% (6.7% to 12.2%); magnesium 28.6% (22.6% to 35.0%); phosphate 19.1% (15.9% to 23.2%); potassium 28.9% (25.8% to 34.6%).

Hospital-level supplementation use varied at least three-fold for all four electrolytes (Fig 3). There was variation in both the overall percentages receiving supplementation per patient-day and the change in supplementation use as levels increased (eFig 5 in S1 Appendix). Median odds ratios ranged from 1.51 (95% CI 1.48–1.55) for calcium (at a calcium level of 8.4 mg/dL), to 2.09 (95% CI 1.99–2.19) for magnesium (at a magnesium level of 2.0 mg/dL) (eTable 5 in S1 Appendix).

At the majority of hospitals, there were no calcium or phosphate levels at which the percentage receiving supplementation exceeded 50% (eTable 6 in S1 Appendix). For magnesium, 75% of hospitals had a magnesium level at which the percentage receiving supplementation was 50% or more, and the range of the maximum levels at which the percentage receiving supplementation was 50% or more was 1.5–2.1 mg/dL. Almost every hospital (96%) had a level of potassium at which the supplementation percentage exceeded 50%, and the range of the maximum such values was 3.3–3.9 mmol/L (eFig 4 in S1 Appendix).

The total cost of supplementation was $2.59 million US dollars, amounting to an average of $53.6 per patient, $15.4 per patient-day, and $28,400 per hospital-year. Median cost per patient-day of supplementation ranged from $6.60 (IQR 1.95 to 17.20) for potassium to $21.80 (IQR 7.80 to 49.50) for calcium.

## Discussion

In this retrospective multicenter cohort study of 47,988 critically ill patients from 52 hospitals in the United States, deficiencies and supplementation of serum calcium, magnesium, phosphate, or potassium were common, and there was substantial hospital-level variability in the use of supplementation. The ubiquity and variability of supplementation highlight an opportunity to improve care for critically ill patients by increasing beneficial supplementation and reducing unnecessary or harmful supplementation.

Our findings of substantial hospital-level variability in supplementation use could be explained by differences in case-mix of critically ill patients. Although we excluded patients with acute or chronic renal failure, hospitals could still have variation in renal function among included patients, with worse renal function potentially leading to lower rates of supplementation [18]. Other relevant case mix variables include baseline nutritional status of critically ill patients [34], proportion of patients presenting with diabetic ketoacidosis [35], or the proportion of patients with atrial fibrillation or other cardiac arrhythmias [36]. Future work could delve deeper into the association between case-mix variables and electrolyte levels and supplementation practices.

Beyond differences in case-mix, differences in hospital- or provider-level practices could also explain observed hospital-level variation in supplementation. However, there is limited evidence to justify using different supplementation practices for different patients. Empiric phosphate replacement was associated with reduced days of mechanical ventilation in a randomized study published in abstract only, and otherwise observational evidence offers conflicting findings on the benefits of and thresholds for phosphate supplementation [10,11,37]. Targeting higher potassium levels did not reduce atrial fibrillation in a randomized trial of patients recovering from cardiac surgery [19]. Targeting higher magnesium levels could be likened to the magnesium prophylaxis shown to reduce new-onset atrial fibrillation in cardiac surgery patients [38]. However, observational evidence suggests that the target to reduce new-onset atrial fibrillation in mixed critically ill patients need not be higher than 0.7 mmol/L (1.7 mg/dL) [36]. The observed variation highlights that it will be beneficial to conduct randomized trials investigating the benefit or harm of supplementation according to patient characteristics.

Our findings corroborate other research showing high prevalences of hypocalcemia [3], hypophosphatemia [1,10,39,40], and hypokalemia [2,9,41,42]. Hypomagnesemia was less common in our study compared to other cohorts of critically ill patients, where hypomagnesemia ranged from 27–66% [1,13,14,43–45]. Exact prevalences of deficiency vary across studies, in part, because they differ in defined lower limits of normal. For example, some argue that the lower limit for magnesium should be 2 mg/dL (0.85 mmol/L) [45,46], and the lower limit of normal for phosphate varied from 0.45 mmol/L to 0.94 mmol/L in a systematic review of observational studies [11]. Reference ranges are generally developed using healthy volunteers [47]; and how these reference ranges apply in critical illness is unclear. Therefore, future work should focus on understanding the impact of supplementation at various electrolyte levels, for critically ill patients with diverse characteristics, on outcomes.

We found that supplementation was not necessarily driven by levels below the normal limits, implying that clinicians may already operate with an implicit critical-care-specific supplementation approach. For example, magnesium supplementation was common at levels well into the normal range, consistent with a belief that higher magnesium levels are beneficial for critically ill patients, while calcium supplementation was uncommon at all ionized calcium levels, consistent with a belief that hypocalcemia is an adaptation to critical illness as opposed to a problem. Other studies of electrolyte supplementation have found that supplementation despite levels within the normal range is common [17,48]. However, the substantial hospital-level variation in supplementation rates that we found suggest that the appropriate thresholds for supplementation remain unclear.

Our study has important limitations. For the electrolytes studied, which are primarily intracellular or stored in tissue, serum levels may not correlate with total body stores [5,6,49,50]. We did not include all electrolytes relevant for critical illness, but instead focused on those commonly treated with discrete supplements administered in a protocolized fashion. Notable omissions include sodium, bicarbonate, chloride, and zinc. We did not analyze calcium, magnesium, phosphate or potassium ingested as food or in enteral feeds, nor did we include this as supplementation. The lower limits of normal for each electrolyte vary. We chose a widely-recognized standard from a national licensing organization in the US, but varying thresholds would change estimated prevalence of deficiency. Calendar days were the unit of sampling, so in some cases supplementation may have preceded the measured electrolyte levels.

We did not analyze the association between deficiencies or supplementation and outcomes such as duration of ventilation or mortality, because it was not possible to account for enough of the relevant confounding variables with the available data. Consequently, our study does not shed light on a central limitation of electrolyte supplementation based on serum levels, which is that variation in serum levels may be only loosely correlated with total body stores, and therefore supplementation based on level might not change clinical outcomes. Although the data are large, they still focus on only a subset of critically ill patients within a single country. We were unable to assess whether observed variation was appropriate, based on the clinical context at the time of each supplementation. We did not attempt to correct for case-mix of critically ill patients or the underlying diagnoses, because the available data were insufficiently detailed to allow for clinically robust adjustment.

Given the heterogeneity of electrolyte supplementation practices, and the complexity of potential interactions between concurrent medications, dietary factors, and other supplements, several types of studies are needed to help improve electrolyte management for critically ill patients. Physiological and observational studies can help clarify the nature of interactions and mechanisms for both benefit and harm with supplementation. Randomized controlled trials can compare the effect of different supplementation practices on clinical outcomes such as arrhythmia, ventilator duration, and survival. The range of supplementation practices described here can help define regions of equipoise. Ideally, trials should gather adequate baseline information to inform pre-specified heterogeneity of treatment analyses, and incorporate cost-effectiveness analysis that helps clinicians, health systems, and policy-makers understand the tradeoffs of different supplementation targets. Observational studies using quasi-experimental or target trial emulation approaches can inform the design of these trials, and guide practice in the interim.

## Conclusion

Deficiency and supplementation of calcium, magnesium, phosphate, and potassium are common in critically ill patients, with substantial variation in supplementation practices across hospitals. Future work should focus on physiological, observational, and randomized studies aimed at distinguishing between beneficial and unnecessary or harmful supplementation, with careful attention to clinical factors that may generate heterogeneity, to improve care for all critically ill patients.

**Key points**

**Question:** Is there hospital-level variation in the supplementation of calcium, magnesium, phosphate, and potassium for critically ill patients?

**Findings:** In this multicentre retrospective cohort study of 47,988 patients from 52 hospitals, 33,626 (70.1%) patients received calcium, magnesium, phosphate, or potassium supplementation. Across hospitals, the median (IQR) percentages receiving supplementation per day were: calcium 9.4% (6.7–12.2); magnesium 28.6% (22.6–35.0); phosphate 19.1% (15.9–23.2); potassium 28.9% (25.8–34.6).

**Meaning:** Supplementation of calcium, magnesium, phosphate, or potassium is common in critically ill patients and varies across hospitals, suggesting a need for randomized trials to clarify optimal supplementation practices.

## Supporting information

S1 Appendix Electronic supplement. This supplement contains the STROBE checklist, additional tables, and additional figures.
(DOCX)
