# Supplementary material for: Supplementation of calcium, magnesium, phosphate, and potassium in critically ill patients: A multicenter cohort study
Source: PLoS One. 2026 Jul 2;21(7):e0349145. doi: 10.1371/journal.pone.0349145 (PMC13327132; doi:10.1371/journal.pone.0349145)
Supplement: S1 Appendix — This supplement contains the STROBE checklist, additional tables, and additional figures. (DOCX) [file pone.0349145.s001.docx]

Electronic supplement: Supplementation of calcium, magnesium, phosphate, and potassium in critically ill patients

2025-11-03

Contents

[1 STROBE Checklist 3](#_Toc224727711)

[2 ICD codes 6](#_Toc224727712)

[2.1 Renal dysfunction 6](#_Toc224727713)

[2.2 Cardiac arrhythmia 6](#_Toc224727714)

[2.3 Fluid and electrolyte disorders 6](#_Toc224727715)

[3 eTable 1: Normal ranges for electrolytes 7](#_Toc224727716)

[4 eTable 2: Major procedures or surgeries on admission 8](#_Toc224727717)

[5 eTable 3: Additional characteristics – US regions 9](#_Toc224727718)

[6 eTable 4: Top 3 supplementation regimens by electrolyte 10](#_Toc224727719)

[7 eTable 5: Median odds ratios showing hospital-level variability in supplementation practices 11](#_Toc224727720)

[8 eTable 6: Electrolyte levels at which supplementation rates exceed 50% 12](#_Toc224727721)

[9 eFigure 1: Flow diagram 13](#_Toc224727722)

[10 eFigure 2: Electrolyte levels over time during ICU admission 14](#_Toc224727723)

[11 eFigure 3: Distribution of electrolyte levels by hospital 15](#_Toc224727724)

[12 eFigure 4: Supplementation by ICU day 16](#_Toc224727725)

[13 eFigure 5: Supplementation probability by electrolyte level and hospital 17](#_Toc224727726)

[14 References 19](#_Toc224727727)

# STROBE Checklist

|  | Item No | Recommendation | Location |
| --- | --- | --- | --- |
| **Title and abstract** | 1 | (*a*) Indicate the study’s design with a commonly used term in the title or the abstract | Abstract p5 |
|  |  | (*b*) Provide in the abstract an informative and balanced summary of what was done and what was found | P5 |
| Introduction | | |  |
| Background/rationale | 2 | Explain the scientific background and rationale for the investigation being reported | Introduction p8 |
| Objectives | 3 | State specific objectives, including any prespecified hypotheses | Introduction p8 |
| Methods | | |  |
| Study design | 4 | Present key elements of study design early in the paper | Methods p9 |
| Setting | 5 | Describe the setting, locations, and relevant dates, including periods of recruitment, exposure, follow-up, and data collection | Methods p9 |
| Participants | 6 | (*a*) Give the eligibility criteria, and the sources and methods of selection of participants. Describe methods of follow-up | Methods p9 |
|  |  | (*b*) For matched studies, give matching criteria and number of exposed and unexposed | N/A |
| Variables | 7 | Clearly define all outcomes, exposures, predictors, potential confounders, and effect modifiers. Give diagnostic criteria, if applicable | Methods p11 |
| Data sources/ measurement | 8* | For each variable of interest, give sources of data and details of methods of assessment (measurement). Describe comparability of assessment methods if there is more than one group | *Methods 10-12* |
| Bias | 9 | Describe any efforts to address potential sources of bias | N/A |
| Study size | 10 | Explain how the study size was arrived at | All available data used |
| Quantitative variables | 11 | Explain how quantitative variables were handled in the analyses. If applicable, describe which groupings were chosen and why | Methods p 10-11 |
| Statistical methods | 12 | (*a*) Describe all statistical methods, including those used to control for confounding | Methods p11 |
|  |  | (*b*) Describe any methods used to examine subgroups and interactions | N/A |
|  |  | (*c*) Explain how missing data were addressed | Methods p10 |
|  |  | (*d*) If applicable, explain how loss to follow-up was addressed | N/A |
|  |  | (*e*) Describe any sensitivity analyses | Methods p11 |
| Results | | |  |
| Participants | 13* | (a) Report numbers of individuals at each stage of study—eg numbers potentially eligible, examined for eligibility, confirmed eligible, included in the study, completing follow-up, and analysed | Results p11, eFigure 1 |
|  |  | (b) Give reasons for non-participation at each stage | eFigure 1 |
|  |  | (c) Consider use of a flow diagram | eFigure 1 |
| Descriptive data | 14* | (a) Give characteristics of study participants (eg demographic, clinical, social) and information on exposures and potential confounders | Results p11 Table 1 |
|  |  | (b) Indicate number of participants with missing data for each variable of interest | N/A |
|  |  | (c) Summarise follow-up time (eg, average and total amount) | Table 2 |
| Outcome data | 15* | Report numbers of outcome events or summary measures over time | Table 2, Results p11-12 |
| Main results | 16 | (*a*) Give unadjusted estimates and, if applicable, confounder-adjusted estimates and their precision (eg, 95% confidence interval). Make clear which confounders were adjusted for and why they were included | Results p12-13 |
|  |  | (*b*) Report category boundaries when continuous variables were categorized | P10 |
|  |  | (*c*) If relevant, consider translating estimates of relative risk into absolute risk for a meaningful time period | Supplement eTable 3 |
| Other analyses | 17 | Report other analyses done—eg analyses of subgroups and interactions, and sensitivity analyses | Supplement eTable 4 |
| Discussion | | |  |
| Key results | 18 | Summarise key results with reference to study objectives | P14 |
| Limitations | 19 | Discuss limitations of the study, taking into account sources of potential bias or imprecision. Discuss both direction and magnitude of any potential bias | P14-16 |
| Interpretation | 20 | Give a cautious overall interpretation of results considering objectives, limitations, multiplicity of analyses, results from similar studies, and other relevant evidence | P14-16 |
| Generalisability | 21 | Discuss the generalisability (external validity) of the study results | P16 |
| Other information | | |  |
| Funding | 22 | Give the source of funding and the role of the funders for the present study and, if applicable, for the original study on which the present article is based | P3 |

*Give information separately for exposed and unexposed groups.

# ICD codes

We identified some variables using combinations of International Classification of Diseases (ICD-10) codes. This was based on prior work.^1,2^

## Renal dysfunction

If any of the following International Classification of Diseases (ICD) codes were present, then we classified a patient as having acute or chronic renal dysfunction: "N17.0", "N17.1", "N17.2", "N17.8", "N17.9", "I13x", "N18x", "N19x", "I12.0x", "N03.2x", "N03.3x", "N03.4x", "N03.5x", "N03.6x", "N03.7x", "N05.2x", "N05.3x", "N05.4x", "N05.5x", "N05.6x", "N05.7x", "N25.0x", "Z49.0x", "Z94.0x", "Z99.2x", "Z39.32x", "Z48.22x", "Z49.31x", "Z91.15x".

## Cardiac arrhythmia

If any of the following International Classification of Diseases (ICD) codes were present, then we classified a patient as having cardiac arrhythmia: "I47x", "I48x", "I49x", "I44.0x", "I44.1x", "I44.3x", "I44.4x", "I44.5x", "I44.6x", "I44.7x", "I44.8x", "I44.9x", "I45.0x", "I45.1x", "I45.2x", "I45.3x", "I45.4x", "I45.5x", "I45.6x", "I45.7x", "I45.8x", "I45.9x", "R00.0x", "R00.1x", "R00.8x", "T82.1x", "Z45.0x", "Z95.0x", "Z95.9x, "Z95.810x", "Z95.818x".

## Fluid and electrolyte disorders

If any of the following ICD codes were present, then we classified a patient as having a fluid and electrolyte disorder: "E86x", "E87x", "E22.2x".

# eTable 1: Normal ranges for electrolytes

*Table: Normal ranges of included electrolytes, from the American Board of Internal Medicine.*^3^

| **Electrolyte (serum)** | **Normal range (units)** | **Normal range (SI units)** |
| --- | --- | --- |
| Calcium – ionized  Calcium – total | 1.12 – 1.23 mmol/L  8.6 – 10.2 mg/dL | Same  2.15 – 2.54 mmol/L |
| Magnesium | 1.6 – 2.6 mg/dL | 0.66 – 1.07 mmol/L |
| Phosphate | 3.0 – 4.5 mg/dL | 0.97 – 1.45 mmol/L |
| Potassium | 3.5 – 5.0 mmol/L | Same |

# eTable 2: Major procedures or surgeries on admission

| **Surgery Type*** | **Count** |
| --- | --- |
| Bypass Coronary Artery, One Artery from Left Internal Mammary, Open Approach | 1377 |
| Replacement of Aortic Valve with Zooplastic Tissue, Open Approach | 883 |
| Dilation of Coronary Artery, One Artery with Drug-eluting Intraluminal Device, Percutaneous Approach | 821 |
| Extirpation of Matter from Intracranial Artery, Percutaneous Approach | 533 |
| Extirpation of Matter from Intracranial Artery using Stent Retriever, Percutaneous Approach | 350 |
| Supplement Mitral Valve with Synthetic Substitute, Open Approach | 319 |
| Replacement of Thoracic Aorta, Ascending/Arch with Synthetic Substitute, Open Approach | 310 |
| Dilation of one coronary artery using two drug-eluting intraluminal devices via a percutaneous approach | 280 |
| Replacement of Mitral Valve with Zooplastic Tissue, Open Approach | 257 |
| Bypass Coronary Artery, One Artery from Aorta with Autologous Venous Tissue, Open Approach | 251 |
| Bypass Coronary Artery, Two Arteries from Aorta with Autologous Venous Tissue, Open Approach | 223 |
| Bypass Trachea to Cutaneous with Tracheostomy Device, Open Approach | 217 |
| Replacement of Aortic Valve with Synthetic Substitute, Open Approach | 203 |
| Replacement of Aortic Valve with Zooplastic Tissue, Percutaneous Approach | 200 |
| Bypass Coronary Artery, Three Arteries from Aorta with Autologous Venous Tissue, Open Approach | 154 |
| Excision of Pancreas, Open Approach | 150 |
| Fusion of 2 to 7 Thoracic Vertebral Joints with Autologous Tissue Substitute, Posterior Approach, Posterior Column, Open Approach | 125 |
| Excision of Cerebral Hemisphere, Open Approach | 120 |
| Dilation of Coronary Artery, One Artery, Percutaneous Approach | 119 |
| Excision of Pituitary Gland, Percutaneous Endoscopic Approach | 109 |

*Based on ICD-10 Procedure Coding System

This table shows the 20 most common major procedures or surgeries on admission, among those who had a major procedure or admission.

# eTable 3: Additional characteristics – US regions

| Characteristic | Total (n=47,988) | Hypocalcemia – total calcium (n=32,925) | Hypocalcemia – ionized calcium (n=7,765) | Hypomagnesemia (n=5,110) | Hypophosphatemia (n=19,201) | Hypokalemia (n=15,213) |
| --- | --- | --- | --- | --- | --- | --- |
| US Census Division, No. (%) |  |  |  |  |  |  |
| East North Central | 11,904 (24.8) | 8107 (24.6) | 2725 (35.1) | 960 (18.8) | 5003 (26.1) | 3664 (24.1) |
| East South Central | 5,032 (10.5) | 3700 (11.2) | 456 (5.9) | 506 (9.9) | 1798 (9.4) | 1571 (10.3) |
| Middle Atlantic | 5,888 (12.3) | 4316 (13.1) | 608 (7.8) | 648 (12.7) | 2270 (11.8) | 1727 (11.4) |
| New England | 3,691 (7.7) | 2238 (6.8) | 795 (10.2) | 455 (8.9) | 1558 (8.1) | 1113 (7.3) |
| Pacific | 697 (1.5) | 550 (1.7) | 53 (0.7) | 98 (1.9) | 412 (2.1) | 293 (1.9) |
| South Atlantic | 12,606 (26.3) | 8548 (26.0) | 2107 (27.1) | 1735 (34.0) | 5393 (28.1) | 4181 (27.5) |
| West North Central | 3,406 (7.1) | 2173 (6.6) | 333 (4.3) | 418 (8.2) | 903 (4.7) | 1008 (6.6) |
| West South Central | 4,764 (9.9) | 3293 (10.0) | 688 (8.9) | 290 (5.7) | 1864 (9.7) | 1656 (10.9) |

Caption: This table shows additional characteristics of included patients, specifically the regions of the hospitals to which they were admitted.

# eTable 4: Top 3 supplementation regimens by electrolyte

| Calcium | Count |
| --- | --- |
| Calcium gluconate parenteral | 11,660 |
| Calcium chloride parenteral | 2,855 |
| Calcium carbonate oral | 2,399 |
| Magnesium |  |
| Magnesium sulfate parenteral | 32,991 |
| Magnesium chloride oral | 5,053 |
| Magnesium chloride parenteral | 41 |
| Phosphate |  |
| Potassium phosphate parenteral | 6,485 |
| Sodium phosphate parenteral | 5,920 |
| Potassium acid phosphate oral | 3,746 |
| Potassium |  |
| Potassium chloride oral | 23,722 |
| Potassium chloride parenteral | 18,431 |
| Potassium bicarbonate oral | 6,598 |

Caption: More than one supplementation regimen could be given per patient-day, such that total supplementations may be more than total count of supplementation regimens patient-days

# eTable 5: Median odds ratios showing hospital-level variability in supplementation practices

| **Electrolyte** | **Level at which median odds ratio was calculated** | **Hospital median odds ratio (95% CI)** | **Approximate observed probability of supplementation per patient-day at that level** | **Absolute increase in probability of supplementation per patient-day implied by median odds ratio** |
| --- | --- | --- | --- | --- |
| Calcium | 8.4mg/dL | 1.81 (1.74, 1.88) | 5% | 3.7% (3.3-4.0) |
| Ionized Calcium | 1.10mmol/L | 1.67 (1.58, 1.78) | 40% | 13% (11-14) |
| Magnesium | 2.0mg/dL | 2.09 (1.99, 2.19) | 15% | 12% (11-13) |
| Phosphate | 3.0mg/dL | 1.95 (1.86, 2.05) | 60% | 15% (14-16) |
| Potassium | 3.9mmol/L | 1.51 (1.48, 1.55) | 30% | 9.3% (8.8-9.9) |

Caption: This table shows the median change in odds when switching from a hospital with a lower rate of supplementation to a hospital with a higher rate of supplementation. For example, when switching from a patient with a serum magnesium level measured at a hospital with a lower rate of supplementation to a patient with the same serum magnesium level measured at a hospital with a higher rate of supplementation, the odds of supplementation would increase by a ratio of 2.09. The approximate observed probabilities of supplementation per patient-day were taken from Figure 2. Absolute increases in probability were calculated by multiplying the approximate probability by the median odds ratio (or its lower / upper bound). Note that the level at which the median odds ratio was calculated was the cohort mean.

# eTable 6: Electrolyte levels at which supplementation rates exceed 50%

| Electrolyte | Number of hospitals where probability of supplementation was 50% or more at one or more levels | Median (IQR) ^2^ | Min, Max |
| --- | --- | --- | --- |
| Calcium | 2 (4%) | 7.8 (7.7, 7.8) | 7.7, 7.8 |
| Ionized Calcium | 20 (38%) | 1.05 (1.05, 1.10) | 1.00, 1.10 |
| Magnesium | 39 (75%) | 1.8 (1.7, 1.9) | 1.5, 2.1 |
| Phosphate | 24 (46%) | 2.4 (2.2, 2.4) | 1.8, 2.6 |
| Potassium | 50 (96%) | 3.50 (3.4, 3.8) | 3.3, 3.9 |

^1^ We excluded hospitals if there was no electrolyte level at which supplementation rates were 50% or more

^2^ Excluding levels with fewer than 50 measurements at that hospital

Recall that the lower limits of normal from the ABIM reference ranges were: total calcium – 8.6mg/dL, ionized calcium – 1.12mmol/L, magnesium – 1.6mg/dL, phosphate – 3.0mg/dL, potassium – 3.5mmol/L.

# eFigure 1: Flow diagram


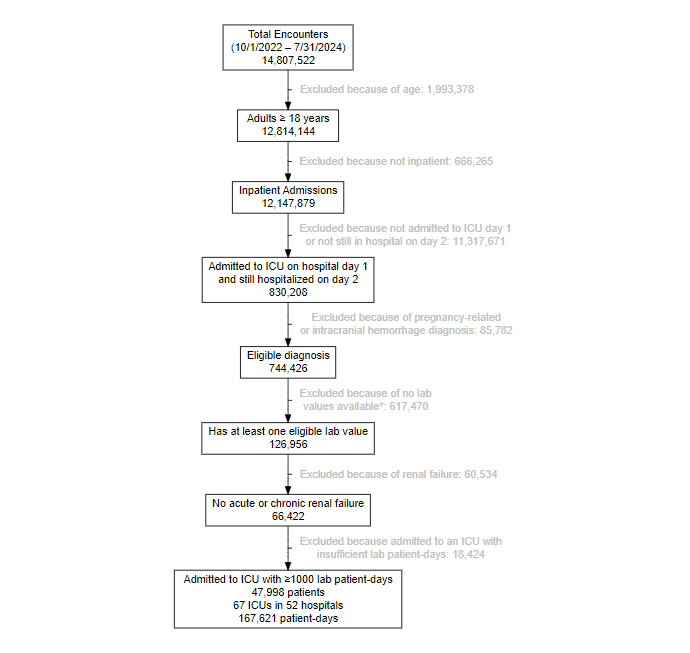


Caption: This flow diagram shows the process by which the study cohort was generated. ICU = intensive care unit. *Note that most patients excluded due to missing laboratory values were excluded because their hospital had no laboratory values available in the database, rather than individual-level missingness.

# eFigure 2: Electrolyte levels over time during ICU admission


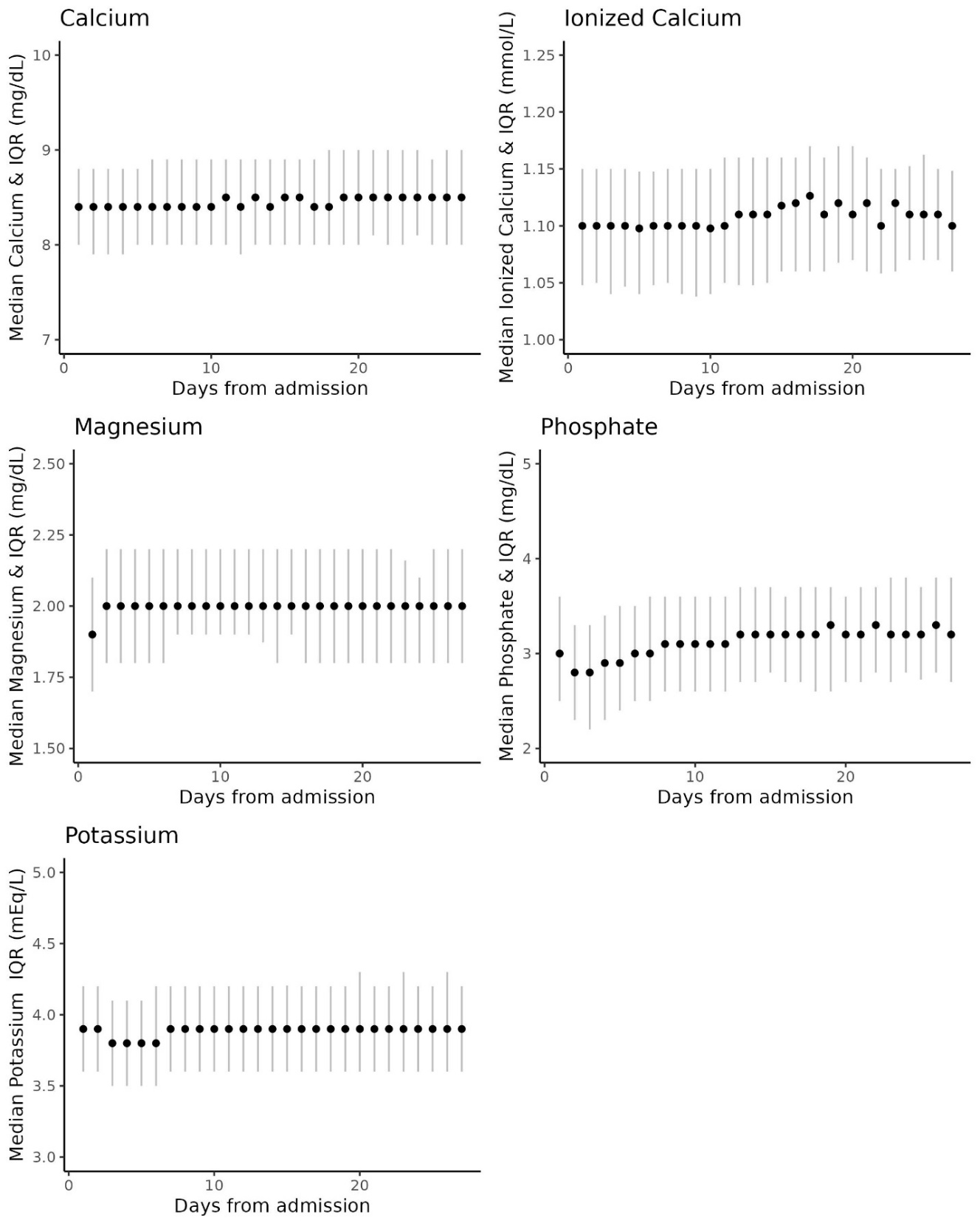


Caption: This figure shows the median and interquartile range of electrolyte levels according to days from intensive care unit (ICU) admission.

# eFigure 3: Distribution of electrolyte levels by hospital


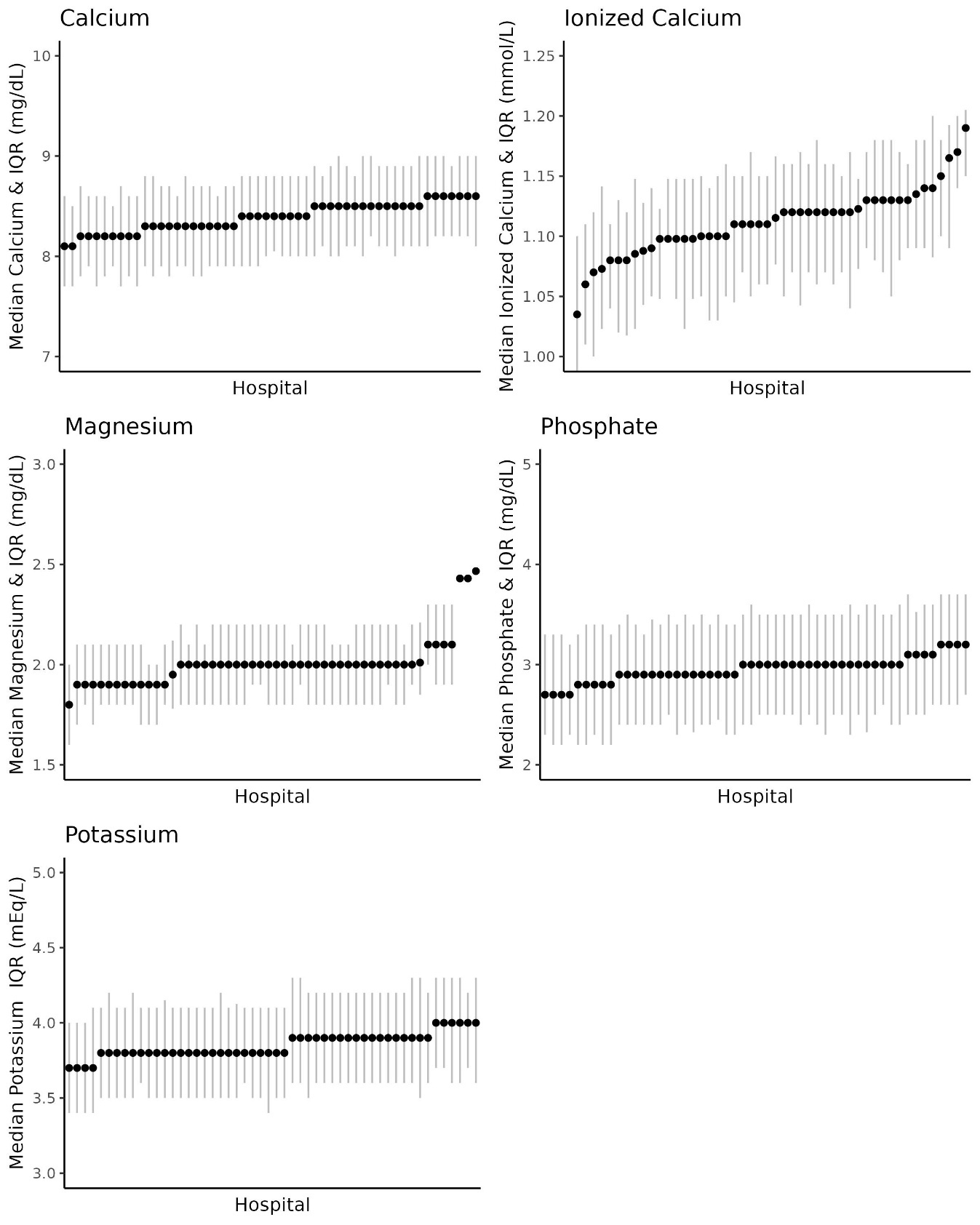


Caption: This five-panel figure shows the distribution of median and interquartile ranges of each electrolyte’s serum levels. Total calcium and ionized calcium are reported separately.

# eFigure 4: Supplementation by ICU day


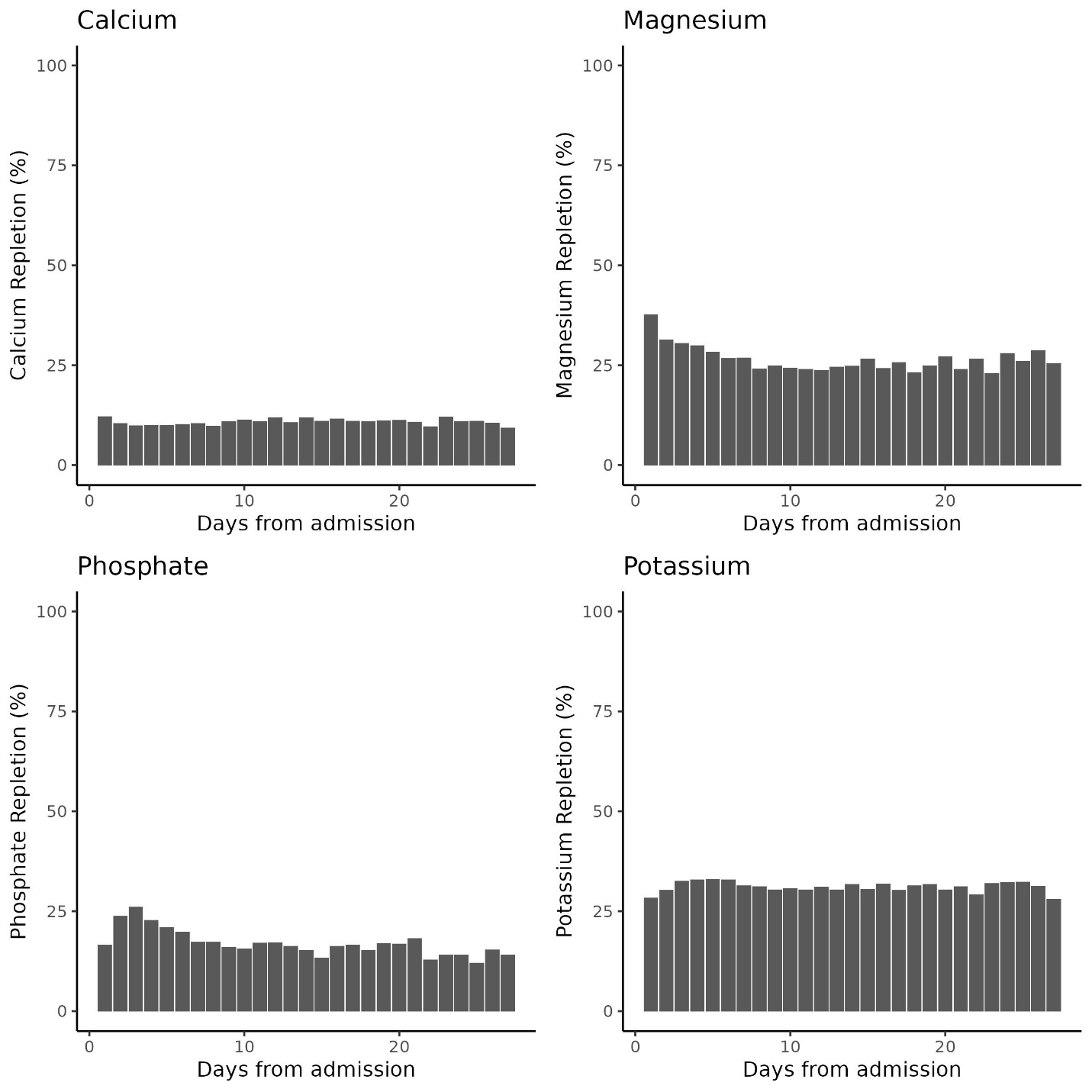


Caption: This four-panel figure shows the percentage of patient-days with a measurement for the corresponding electrolyte, according to the date relative to admission when the measurement was taken. Note that either ionized or total calcium levels were considered a patient-day in the denominator for the “Calcium” panel. Across the four electrolytes, the medians (interquartile ranges) of supplementation probabilities across days were: calcium 10.9% (10.3% to 11.2%); magnesium 25.7% (24.3% to 27.5%); phosphate 16.6% (15.2% to 17.3%); potassium 31.2% (30.3% to 31.9%).

# eFigure 5: Supplementation probability by electrolyte level and hospital


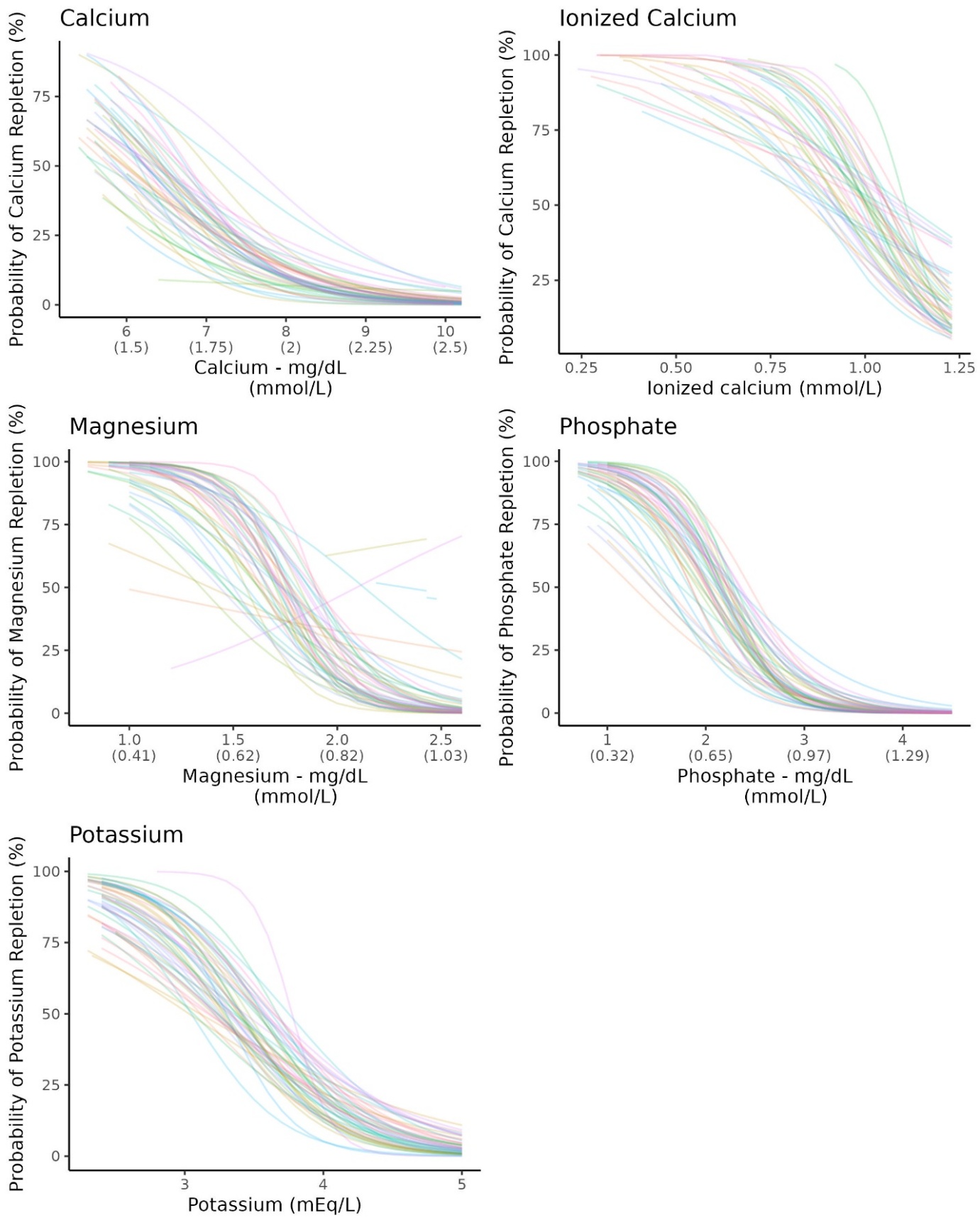


Caption: This figure shows the predicted probability of supplementation (y-axis) according to serum electrolyte level (x-axis). Predicted probabilities were generated from the multilevel logistic regression model with hospital-level random intercepts and slopes. Each hospital appears as a separate thin coloured line. This shows the similarities and differences across hospitals in supplementation practices, both with respect to the absolute electrolyte levels that prompt supplementation (position of the curve on the x-axis) and the extent to which a particular threshold may be driving behaviour (steepness of the transition from high to low probabilities of supplementation).

# References

1. Sun JW, Rogers JR, Her Q, et al. Adaptation and Validation of the Combined Comorbidity Score for ICD-10-CM. *Med Care*. 2017;55(12):1046-1051. doi:10.1097/MLR.0000000000000824

2. Bosch NA, Law AC, Rucci JM, Peterson D, Walkey AJ. Predictive Validity of the Sequential Organ Failure Assessment Score versus Claims-based Scores among Critically Ill Patients. *Ann Am Thorac Soc*. 19(6):1072-1076. doi:10.1513/AnnalsATS.202111-1251RL

3. ABIM Laboratory Test Reference Ranges - January 2024. American Board of Internal Medicine. January 2024. Accessed June 20, 2024. https://www.abim.org/certification/exam-information/internal-medicine/reference-ranges
