# Supplementary material for: Supplementation of calcium, magnesium, phosphate, and potassium in critically ill patients: A multicenter cohort study
Source: PLoS One. 2026 Jul 2;21(7):e0349145. doi: 10.1371/journal.pone.0349145 (PMC13327132; doi:10.1371/journal.pone.0349145)
Supplement: Attachment — Submitted filename: CaMgKPhos Plos One Response to Reviewers R1.docx [file pone.0349145.s003.docx]

Dear Editor Dr Mohammed S. Razzaque and the PLOS One Editorial Team,

Thank you for the opportunity to revise our manuscript, “Supplementation of calcium, magnesium, phosphate, and potassium in critically ill patients: a multicenter cohort study.” We have addressed all editorial and reviewer comments (*numbered in italics)*, with responses in this letter (additions in **blue**), and have uploaded tracked-changes and clean versions of the manuscript, along with figures, tables, and supplement, to the journal submission website.

**Editorial comments**

1. *Please ensure that your manuscript meets PLOS ONE's style requirements, including those for file naming.*

We have adjusted formatting to ensure we meet the style requirements.

2. *According to your manuscript, your waiver of ethical approval was secured from the Boston University Institutional Review Board (#H-41795). However, your wavier of ethical approval document has a different reference number (H-43331). Please provide the corrected reference number in your manuscript or provide an explanation for this discrepancy.*

The correct number is H-43331 and the manuscript and attachment now match.

3. *We note that you have indicated that there are restrictions to data sharing for this study. For studies involving human research participant data or other sensitive data, we encourage authors to share de-identified or anonymized data. However, when data cannot be publicly shared for ethical reasons, we allow authors to make their data sets available upon request. Before we proceed with your manuscript, please address the following prompts: a) If there are ethical or legal restrictions on sharing a de-identified data set, please explain them in detail (e.g., data contain potentially identifying or sensitive patient information, data are owned by a third-party organization, etc.) and who has imposed them (e.g., a Research Ethics Committee or Institutional Review Board, etc.). Please also provide contact information for a data access committee, ethics committee, or other institutional body to which data requests may be sent.*

There are legal restrictions on sharing the data because they are owned by a third-party organization (Premier Inc), not the authors, and therefore the authors are not able to share the data. The database is accessible through Premier Inc (<https://premierinc.com/real-world-evidence/real-world-data>). Premier Inc charges for access to the database.

4. *Please update your Data Availability statement in the submission form accordingly.*

We have updated the Data Availability statement which now reads:

“The data use agreement between Premier Inc. and Boston University prevents sharing of the datasets used for this study. **There are legal restrictions on sharing the data because they are owned by a third-party organization (Premier Inc), not the authors, and therefore the authors are not able to share the data. The database is accessible through Premier Inc (https://premierinc.com/real-world-evidence/real-world-data). Premier Inc charges for access to the database.** Analytic code is available on the Open Science Framework (https://osf.io/ndjyh/overview).”

5. *Please include captions for your Supporting Information files at the end of your manuscript, and update any in-text citations to match accordingly. Please see our Supporting Information guidelines for more information:* [*http://journals.plos.org/plosone/s/supporting-information*](http://journals.plos.org/plosone/s/supporting-information)*.*

We have added a caption to “S1 Appendix”, updated in-text citations to match, and adjusted the file name.

**Reviewer 1 comments:**

*Major comments.*

6. *The manuscript does not adequately address the underlying causes of abnormal electrolyte levels (e.g., sepsis, gastrointestinal losses, diuretic use, nutritional status, acid–base disorders). Without this context, it is difficult to interpret whether observed supplementation practices reflect appropriate responses to reversible conditions or empiric replacement. Can the authors discuss whether such factors were available in the database?*

The reviewer is correct that our manuscript does not focus on the underlying causes of abnormal electrolyte levels. The Premier Healthcare Database does not have sufficient granularity about the time-sequence or nuance of the diagnoses that lead to electrolyte deficiency for these to be incorporated in the analysis. Even with that data, accepted clinical practice around electrolyte supplementation is often based on eminence rather than evidence, and so it would be difficult to know whether supplementation prompted by a given clinical scenario was beneficial or not. We have expanded our limitations section by adding the following:

“We did not attempt to correct for case-mix of critically ill patients **or the underlying diagnoses, because the available data were insufficiently detailed to allow for clinically robust adjustment.**”

7. *Patients with acute or chronic renal failure were excluded, but the definitions are not sufficiently detailed.*

If patients had a diagnosis of acute or chronic renal dysfunction based on their International Classification of Disease codes, then we excluded them. We have updated the Patients sub-section of the Methods section which now reads:

“We excluded patients with diagnoses of acute or chronic renal dysfunction **(Section 2 in Appendix S1)** because electrolyte supplementation may be less common in such patients; patients with a pregnancy-related diagnosis (ICD-10 O00-O9A) because of magnesium administration for pre-eclampsia(23); and patients with intracranial bleeding because some centers may administer magnesium to aid in cerebral hemostasis.”

And in Appendix S1 we now include a section reading: “**If any of the following International Classification of Diseases (ICD) codes were present, then we classified a patient as having acute or chronic renal dysfunction: "N17.0", "N17.1", "N17.2", "N17.8", "N17.9", "I13x", "N18x", "N19x", "I12.0x", "N03.2x", "N03.3x", "N03.4x", "N03.5x", "N03.6x", "N03.7x", "N05.2x", "N05.3x", "N05.4x", "N05.5x", "N05.6x", "N05.7x", "N25.0x", "Z49.0x", "Z94.0x", "Z99.2x", "Z39.32x", "Z48.22x", "Z49.31x", "Z91.15x".**”

8. *The manuscript references mechanical ventilation in the discussion, yet the proportion of patients receiving mechanical ventilation in the cohort is not reported. Can the authors look at the frequency of mechanical ventilation?*

We have added this information to Table 1. A total of 16,183 (33.6%) were receiving invasive mechanical ventilation on admission.

9. *The cohort likely includes a heterogeneous mix of medical and surgical ICU patients. Please look at medical vs surgical admissions, and the number of patients undergoing major surgeries, such as cardiac or abdominal surgery*

We have added a row showing the proportion of patients who had a major procedure or surgery on admission to Table 1 (15,992, 33.2%), and we have added information about surgical admissions to new supplemental table now numbered eTable 1 in S1 Appendix.

10. *Can the authors please clarify whether admissions were elective or emergent?*

The data include all patients admitted to the intensive care unit who had the studied electrolytes sampled. This includes both elective and emergent admissions.

11. *Sodium is a commonly monitored and clinically important electrolyte in ICU patients. Can the authors justify why sodium was excluded from the analysis?*

Sodium was not included because it is generally not supplemented based on the serum level, nor is it commonly an element of electrolyte supplementation protocols. We have added this explanation to the Methods section:

“**We focused on levels and supplementation of calcium, magnesium, phosphate, and potassium, because these electrolytes are commonly measured in critically ill patients and commonly included in serum level-based supplementation protocols ordered at ICU admission. W** **We did not include other electrolytes such as sodium or zinc, which are uncommonly supplemented in a protocolized fashion.**”

12. *It is unclear why congestive heart failure (CHF) was not included among reported comorbidities, given its association with electrolyte abnormalities and supplementation decisions.*

We have added congestive heart failure to the list of comorbidities. A total of 13,764 (28.7%) had congestive heart failure.

13. *Related to this, how the term “cardiac arrhythmia” in table 1 was identified?*

Cardiac arrhythmia was defined using ICD codes. If any of the following codes were present, we classified the patient as having cardiac arrhythmia. This was based on prior work now cited in the manuscript (10.1513/AnnalsATS.202111-1251RL, 10.1097/MLR.0000000000000824): "I47x", "I48x", "I49x", "I44.0x", "I44.1x", "I44.3x", "I44.4x", "I44.5x", "I44.6x", "I44.7x", "I44.8x", "I44.9x", "I45.0x", "I45.1x", "I45.2x", "I45.3x", "I45.4x", "I45.5x", "I45.6x", "I45.7x", "I45.8x", "I45.9x", "R00.0x", "R00.1x", "R00.8x", "T82.1x", "Z45.0x", "Z95.0x", "Z95.9x, "Z95.810x", "Z95.818x".

14. *The manuscript lists “fluid and electrolyte disorders” as a comorbidity in table 1. Please clarify: 1) How this variable was defined (ICD codes?),*

This variable included the following ICD codes: "E86x", "E87x", "E22.2x" and this is now explained in Section 2, S1 Appendix.

15. *2) Whether it overlaps with the electrolyte abnormalities measured in this study, and*

Yes, we anticipate that there may have been overlap with electrolyte abnormalities seen on admission in patients included in this study.

16. *3) Whether inclusion of this variable risks circular reasoning or misclassification bias.*

If our work had involved adjusting for baseline variables such as diagnosis or other clinical details, then there is a possibility that including redundant information (such as a diagnosis of fluid or electrolyte abnormalities) could introduce collider bias. We did not build any models to adjust for baseline characteristics, because our main goals were to describe frequency and its variation, within the limitations of unadjusted assessment.

**Reviewer 2 comments:**

17. *The manuscript provides valuable descriptive data on electrolyte supplementation practices across U.S. ICUs, but its conceptual framing is limited by a narrow focus on serum values*

We agree that serum levels of electrolytes do not necessarily correlate with total body stores, especially for electrolytes which are primarily found intracellularly or in tissues (e.g. calcium, magnesium, phosphate, potassium). We have updated the limitations section:

“Our study has important limitations. **For the electrolytes studied, which are primarily intracellular or stored in tissue, serum levels are not necessarily correlated with total body stores.(5, 6, 49, 50)** Calendar days…”

18. *and it is not clear how supplementation was adminstered,*

The top 3 supplementation regimens for each electrolyte are available in eTable 3 in the electronic supplement. We have made this more clear in the manuscript:

“The most common supplementation route was parenteral for calcium, magnesium, and phosphate, and oral for potassium (eTable 3 in S1 Appendix **contains additional details about administered supplements**).”

19. *nor if it included dietician prescribed medical nutrition therapy.*

We did not include electrolytes included in hospital food or enteral nutrition formulations ordered by the dietitian, and have added this to the limitations:

“**We did not analyze calcium, magnesium, phosphate or potassium ingested as food or in enteral feeds, nor did we include this as supplementation.**”

20. *The study does not provide sufficient details of the role of physiologic mechanisms that drive electrolyte abnormalities in critical illness,*
21. *nor does it acknowledge that serum concentrations are poor proxies for tissue concentrations. For example, hypophosphatemia is common in hospitalized patients due to a shift of phosphate into tissue.*

Please see response to comment 17.

22. *Notably, the omission of sodium — the most commonly administered electrolyte and a major driver of iatrogenic harm from intravenous saline — further limits the comprehensiveness of the analysis. Indeed, hyponatremia is often misdiagnosed in hypervolemic conditions in which sodium concentrations are diluted by excessive fluid retention.*

Please see response to comment 11.

23. *The complex interaction of sodium levels with hypokalemia is also important.*

Thank you for flagging this point. We do not feel it is possible to sufficiently address it within the conceptual and logistical space of this manuscript, but we have added the following to the future directions identified at the end:

“Future work should focus on **observational and randomized studies** aimed at distinguishing between beneficial and unnecessary **or harmful** supplementation, **with careful attention to clinical factors that may generate heterogeneity,** to improve care for all critically ill patients.”

24. *Furthermore, the paper treats supplementation as benign and does not sufficiently focus on their potential adverse effects associated with aggressive dosages.*

We agree that supplementation is not necessarily benign, and part of our overall goal in performing this work was to call into question the benefit of ubiquitous supplementation. We have revised the start of the discussion section which now reads: “The ubiquity and variability of supplementation highlight an opportunity to improve care for critically ill patients by increasing beneficial supplementation and reducing unnecessary **or harmful** supplementation.”, and the last sentence of the manuscript (see response to comment 23).

25. *Overall, the paper would benefit from a more physiologically grounded discussion of the well-documented pathophysiological causes related to electrolyte dysregulation and a clearer acknowledgment that supplementation practices only superficially target serum numbers rather than underlying pathophysiology. Such a grounded discussion could eventually contribute to correcting the identified inconsistencies in supplementation practices in ICUs.*
26. *Finally, the authors call for randomized controlled trials, but such clinical designs make little sense without a more comprehensive understanding of the underlying causative factors that disturb electrolyte homeostasis in critically ill patients.*

Our perspective in calling for randomized trials is that this is the study design that will minimize bias from confounding by indication and will generate the strength of evidence needed to change practice. We have left in the call for randomization but added observational studies as well: “Future work should focus on **observational and randomized studies** aimed at distinguishing between beneficial and unnecessary **or harmful** supplementation, **with careful attention to clinical factors that may generate heterogeneity,** to improve care for all critically ill patients.”

Thank you for the opportunity to revise and resubmit.

Sincerely,

Christopher Yarnell MD PhD
