# Supplementary material for: Supplementation of calcium, magnesium, phosphate, and potassium in critically ill patients: A multicenter cohort study
Source: PLoS One. 2026 Jul 2;21(7):e0349145. doi: 10.1371/journal.pone.0349145 (PMC13327132; doi:10.1371/journal.pone.0349145)
Supplement: Attachment — Submitted filename: CaMgKPhos Plos One Response to Reviewers R2.docx [file pone.0349145.s004.docx]

Dear Editor Dr Mohammed S. Razzaque and the PLOS One Editorial Team,

Thank you for the opportunity to revise our manuscript, “Supplementation of calcium, magnesium, phosphate, and potassium in critically ill patients: a multicenter cohort study.” We have addressed all editorial and reviewer comments (*numbered in italics)*, with responses in this letter (additions in **blue**), and have uploaded tracked-changes and clean versions of the manuscript, along with figures, tables, and supplement, to the journal submission website.

**Editorial comments:**

*1. Please explain why sodium assessment was not included in the study and highlight it as a potential limitation.*

We did not include sodium assessment in the study because sodium is not an electrolyte that is commonly treated with discrete supplementation in a protocolized fashion according to serum levels in critically ill patients. Hyponatremia treatment sometimes involves sodium supplementation, when the etiology of the hyponatremia is inappropriate antidiuretic hormone activity and fluid restriction alone does not suffice. Description of the epidemiology of sodium and its supplementation would be a worthwhile project for a separate manuscript. Other electrolytes that we did not discuss in this work include chloride, bicarbonate, and zinc.

We adjusted the following sentence in the Methods:

“We did not include other electrolytes such as sodium, **chloride, bicarbonate,** or zinc, which are uncommonly supplemented in a protocolized fashion.”

We added the following limitation to the discussion:

“**We did not include all electrolytes relevant for critical illness, but instead focused on those commonly treated with** **discrete supplements administered in a protocolized fashion. Notable omissions include sodium, bicarbonate, chloride, and zinc.”**

**Reviewer 1 comments:**

*2. Reviewer #1: I appreciate the authors’ explanation for excluding sodium; however, I remain concerned that this omission limits the completeness of a descriptive study of electrolyte management in the ICU. Hyponatremia is common and clinically important in critically ill patients, and its correction—particularly in moderate to severe cases—reflects active management decisions and likely inter‑hospital variation, even if not protocolized in the same manner as other electrolytes. Inclusion of sodium would enhance the clinical relevance of the study.*

Please see response to editor comment 1. Description of the epidemiology of sodium and its supplementation, including investigation of inter-hospital variation, would be a worthwhile project for a separate manuscript. Sodium supplementation intended to expand the intravascular volume is different from protocolized supplementation of calcium, magnesium, phosphate, or potassium based on level. Sodium supplementation for hyponatremia has separate nuances around rate of correction and extravascular fluid status (hyper- or hypovolemic), which are distinct from the nuances of the studied electrolytes.

**Reviewer 2 comments:**

*3. Reviewer #2: Regarding sodium in Comment 11: The authors' rationale for excluding sodium requires correction. The manuscript states: “Sodium was not included because it is generally not supplemented based on the serum level…” This is inaccurate. In ICU practice, serum sodium is routinely monitored and sodium is supplemented accordingly—most commonly through intravenous fluids such as normal saline. Thus, the current wording implies a misunderstanding of standard electrolyte management. Please revise to clarify that sodium was excluded not because it is rarely supplemented, but because its supplementation is embedded within fluid management rather than ordered as a discrete electrolyte replacement, and therefore falls outside the scope of the present analysis of discretionary supplementation.*

Thank you for this point. We agree that sodium supplementation is common but distinct from the supplementation used for the studied electrolytes. Please see response to Editor comment 1 for further details and our addition to the Limitations section.

*4. Regarding Comment 23: The response does not fully address the broader point that electrolyte supplementation in ICU patients is influenced by interactions with other supplements, dietary factors, and concurrent medications. These interactions can generate clinical harm or interfere with medical treatments. The manuscript should note that future studies should examine these supplement interactions, as they represent a potential source of adverse effects in electrolyte management.*

We have adjusted the final paragraph of the discussion (just prior to the conclusion), which now reads:

“Given the heterogeneity of electrolyte supplementation practices, **and the complexity of potential interactions between concurrent medications, dietary factors, and other supplements, several types of studies are needed to help improve electrolyte management for critically ill patients. Physiological and observational studies can help clarify the nature of interactions and mechanisms for both benefit and harm with supplementation.** Randomized controlled trials can compare…”

*5. Regarding Comment 25: The authors’ made no response to acknowledge a central limitation of electrolyte supplementation which often treats superficial serum fluctuations without addressing the underlying homeostatic disturbances that generate them. A brief acknowledgment of this limitation is needed along with a call for further investigations of pathophysiology mechanisms associated with electrolyte dysregulation. Clinical trials and observational studies alone cannot address the need for basic research in this area.*

We agree that serum fluctuations do not necessarily correlate with underlying deficiency and therefore the entire enterprise of level-based electrolyte supplementation may be flawed. We have added the following to the Limitations paragraph:

“We did not analyze the association between deficiencies or supplementation and outcomes such as duration of ventilation or mortality, because it was not possible to account for enough of the relevant confounding variables with the available data. **Consequently, our study does not shed light on a central limitation of electrolyte supplementation based on serum levels, which is that variation in serum levels may be only loosely correlated with total body stores, and therefore supplementation based on level might not change clinical outcomes.**”

We also made an addition to the final Conclusions paragraph:

“Future work should focus on **physiological,** observational, and randomized studies aimed at distinguishing between beneficial and unnecessary or harmful supplementation, with careful attention to clinical factors that may generate heterogeneity, to improve care for all critically ill patients.”

We also mentioned physiological studies in the future directions paragraph, as outlined in response to reviewer comment 4 above.

Thank you for the opportunity to revise and resubmit.

Sincerely,

Christopher Yarnell MD PhD
