# Supplementary material for: Supplementation of calcium, magnesium, phosphate, and potassium in critically ill patients: A multicenter cohort study
Source: PLoS One. 2026 Jul 2;21(7):e0349145. doi: 10.1371/journal.pone.0349145 (PMC13327132; doi:10.1371/journal.pone.0349145)
Supplement: Peer review history

# Peer review: Decision Letter 0

**DOI:** 10.1371/journal.pone.0349145.r001

**Authors:** Mohammed S. Razzaque

8 Feb 2026

PONE-D-26-00828

Supplementation of calcium, magnesium, phosphate, and potassium in critically ill patients: a multicenter cohort study

PLOS One

Dear Dr. Yarnell,

Thank you for submitting your manuscript to PLOS ONE. After careful consideration, we feel that it has merit but does not fully meet PLOS ONE’s publication criteria as it currently stands. Therefore, we invite you to submit a revised version of the manuscript that addresses the points raised during the review process.

Please submit your revised manuscript by Mar 25 2026 11:59PM. If you will need more time than this to complete your revisions, please reply to this message or contact the journal office at plosone@plos.org. When you're ready to submit your revision, log on to https://www.editorialmanager.com/pone/ and select the 'Submissions Needing Revision' folder to locate your manuscript file.

Please include the following items when submitting your revised manuscript:

- A letter that responds to each point raised by the academic editor and reviewer(s). You should upload this letter as a separate file labeled 'Response to Reviewers'.
- A marked-up copy of your manuscript that highlights changes made to the original version. You should upload this as a separate file labeled 'Revised Manuscript with Track Changes'.
- An unmarked version of your revised paper without tracked changes. You should upload this as a separate file labeled 'Manuscript'.

If you would like to make changes to your financial disclosure, please include your updated statement in your cover letter. Guidelines for resubmitting your figure files are available below the reviewer comments at the end of this letter.

If applicable, we recommend that you deposit your laboratory protocols in protocols.io to enhance the reproducibility of your results. Protocols.io assigns your protocol its own identifier (DOI) so that it can be cited independently in the future. For instructions see: https://journals.plos.org/plosone/s/submission-guidelines#loc-laboratory-protocols. Additionally, PLOS ONE offers an option for publishing peer-reviewed Lab Protocol articles, which describe protocols hosted on protocols.io. Read more information on sharing protocols at https://plos.org/protocols?utm_medium=editorial-email&utm_source=authorletters&utm_campaign=protocols.

We look forward to receiving your revised manuscript.

Kind regards,

Mohammed S. Razzaque, MBBS, PhD

Academic Editor

PLOS One

Journal Requirements:

When submitting your revision, we need you to address these additional requirements.

1. Please ensure that your manuscript meets PLOS ONE's style requirements, including those for file naming. The PLOS ONE style templates can be found at

https://journals.plos.org/plosone/s/file?id=wjVg/PLOSOne_formatting_sample_main_body.pdf and

https://journals.plos.org/plosone/s/file?id=ba62/PLOSOne_formatting_sample_title_authors_affiliations.pdf

2. According to your manuscript, your waiver of ethical approval was secured from the Boston University Institutional Review Board (#H-41795). However, your wavier of ethical approval document has a different reference number (H-43331). Please provide the corrected reference number in your manuscript or provide an explanation for this discrepancy.

3. We note that you have indicated that there are restrictions to data sharing for this study. For studies involving human research participant data or other sensitive data, we encourage authors to share de-identified or anonymized data. However, when data cannot be publicly shared for ethical reasons, we allow authors to make their data sets available upon request. For information on unacceptable data access restrictions, please see http://journals.plos.org/plosone/s/data-availability#loc-unacceptable-data-access-restrictions.

Before we proceed with your manuscript, please address the following prompts:

a) If there are ethical or legal restrictions on sharing a de-identified data set, please explain them in detail (e.g., data contain potentially identifying or sensitive patient information, data are owned by a third-party organization, etc.) and who has imposed them (e.g., a Research Ethics Committee or Institutional Review Board, etc.). Please also provide contact information for a data access committee, ethics committee, or other institutional body to which data requests may be sent.

b) If there are no restrictions, please upload the minimal anonymized data set necessary to replicate your study findings to a stable, public repository and provide us with the relevant URLs, DOIs, or accession numbers. Please see http://www.bmj.com/content/340/bmj.c181.long for guidelines on how to de-identify and prepare clinical data for publication. For a list of recommended repositories, please see https://journals.plos.org/plosone/s/recommended-repositories. You also have the option of uploading the data as Supporting Information files, but we would recommend depositing data directly to a data repository if possible.

Please update your Data Availability statement in the submission form accordingly.

4. Please include captions for your Supporting Information files at the end of your manuscript, and update any in-text citations to match accordingly. Please see our Supporting Information guidelines for more information: http://journals.plos.org/plosone/s/supporting-information.

5. If the reviewer comments include a recommendation to cite specific previously published works, please review and evaluate these publications to determine whether they are relevant and should be cited. There is no requirement to cite these works unless the editor has indicated otherwise.

[Note: HTML markup is below. Please do not edit.]

Reviewers' comments:

Reviewer's Responses to Questions

**Comments to the Author**

1. Is the manuscript technically sound, and do the data support the conclusions?

The manuscript must describe a technically sound piece of scientific research with data that supports the conclusions. Experiments must have been conducted rigorously, with appropriate controls, replication, and sample sizes. The conclusions must be drawn appropriately based on the data presented.

Reviewer #1: Yes

Reviewer #2: Yes

**********

2. Has the statistical analysis been performed appropriately and rigorously?

Reviewer #1: Yes

Reviewer #2: Yes

**********

3. Have the authors made all data underlying the findings in their manuscript fully available?

The PLOS Data policy requires authors to make all data underlying the findings described in their manuscript fully available without restriction, with rare exception (please refer to the Data Availability Statement in the manuscript PDF file). The data should be provided as part of the manuscript or its supporting information, or deposited to a public repository. For example, in addition to summary statistics, the data points behind means, medians and variance measures should be available. If there are restrictions on publicly sharing data—e.g. participant privacy or use of data from a third party—those must be specified.

Reviewer #1: Yes

Reviewer #2: No

**********

4. Is the manuscript presented in an intelligible fashion and written in standard English?

PLOS ONE does not copyedit accepted manuscripts, so the language in submitted articles must be clear, correct, and unambiguous. Any typographical or grammatical errors should be corrected at revision, so please note any specific errors here.

Reviewer #1: Yes

Reviewer #2: Yes

**********

5. Review Comments to the Author

Please use the space provided to explain your answers to the questions above. You may also include additional comments for the author, including concerns about dual publication, research ethics, or publication ethics. (Please upload your review as an attachment if it exceeds 20,000 characters)

Reviewer #1: Thank you for the opportunity to review the manuscript, entitled “Supplementation of calcium, magnesium, phosphate, and potassium in critically ill patients: a multicenter cohort study.” This manuscript addresses an important issue in critical care: hospital-level variation in electrolyte supplementation practices among critically ill patients. However, several clinical and methodological issues require clarification to improve interpretability and clinical relevance. I have the following questions and comments to the authors.

Major comments.

• The manuscript does not adequately address the underlying causes of abnormal electrolyte levels (e.g., sepsis, gastrointestinal losses, diuretic use, nutritional status, acid–base disorders). Without this context, it is difficult to interpret whether observed supplementation practices reflect appropriate responses to reversible conditions or empiric replacement. Can the authors discuss whether such factors were available in the database?

• Patients with acute or chronic renal failure were excluded, but the definitions are not sufficiently detailed.

• The manuscript references mechanical ventilation in the discussion, yet the proportion of patients receiving mechanical ventilation in the cohort is not reported. Can the authors look at the frequency of mechanical ventilation?

• The cohort likely includes a heterogeneous mix of medical and surgical ICU patients. Please look at medical vs surgical admissions, and the number of patients undergoing major surgeries, such as cardiac or abdominal surgery

• Can the authors please clarify whether admissions were elective or emergent?

• Sodium is a commonly monitored and clinically important electrolyte in ICU patients. Can the authors justify why sodium was excluded from the analysis?

• It is unclear why congestive heart failure (CHF) was not included among reported comorbidities, given its association with electrolyte abnormalities and supplementation decisions. Related to this, how the term “cardiac arrhythmia” in table 1 was identified?

• The manuscript lists “fluid and electrolyte disorders” as a comorbidity in table 1. Please clarify: 1) How this variable was defined (ICD codes?), 2) Whether it overlaps with the electrolyte abnormalities measured in this study, and 3) Whether inclusion of this variable risks circular reasoning or misclassification bias.

Reviewer #2: The manuscript provides valuable descriptive data on electrolyte supplementation practices across U.S. ICUs, but its conceptual framing is limited by a narrow focus on serum values and it is not clear how supplementation was adminstered, nor if it included dietician prescribed medical nutrition therapy. The study does not provide sufficient details of the role of physiologic mechanisms that drive electrolyte abnormalities in critical illness, nor does it acknowledge that serum concentrations are poor proxies for tissue concentrations. For example, hypophosphatemia is common in hospitalized patients due to a shift of phosphate into tissue. Notably, the omission of sodium — the most commonly administered electrolyte and a major driver of iatrogenic harm from intravenous saline — further limits the comprehensiveness of the analysis. Indeed, hyponatremia is often misdiagnosed in hypervolemic conditions in which sodium concentrations are diluted by excessive fluid retention. The complex interaction of sodium levels with hypokalemia is also important. Furthermore, the paper treats supplementation as benign and does not sufficiently focus on their potential adverse effects associated with aggressive dosages. Overall, the paper would benefit from a more physiologically grounded discussion of the well-documented pathophysiological causes related to electrolyte dysregulation and a clearer acknowledgment that supplementation practices only superficially target serum numbers rather than underlying pathophysiology. Such a grounded discussion could eventually contribute to correcting the identified inconsistencies in supplementation practices in ICUs. Finally, the authors call for randomized controlled trials, but such clinical designs make little sense without a more comprehensive understanding of the underlying causative factors that disturb electrolyte homeostasis in critically ill patients.

**********

6. PLOS authors have the option to publish the peer review history of their article (what does this mean?). If published, this will include your full peer review and any attached files.

If you choose “no”, your identity will remain anonymous but your review may still be made public.

**Do you want your identity to be public for this peer review?**  For information about this choice, including consent withdrawal, please see our Privacy Policy.

Reviewer #1: No

Reviewer #2: No

**********

[NOTE: If reviewer comments were submitted as an attachment file, they will be attached to this email and accessible via the submission site. Please log into your account, locate the manuscript record, and check for the action link "View Attachments". If this link does not appear, there are no attachment files.]

To ensure your figures meet our technical requirements, please review our figure guidelines: https://journals.plos.org/plosone/s/figures

You may also use PLOS’s free figure tool, NAAS, to help you prepare publication quality figures: https://journals.plos.org/plosone/s/figures#loc-tools-for-figure-preparation.

NAAS will assess whether your figures meet our technical requirements by comparing each figure against our figure specifications.

# Author comment: Author response to Decision Letter 1

**DOI:** 10.1371/journal.pone.0349145.r002

26 Mar 2026

Please see attached response to reviewers letter as well. Thanks for the opportunity to revise.

Dear Editor Dr Mohammed S. Razzaque and the PLOS One Editorial Team,

Thank you for the opportunity to revise our manuscript, “Supplementation of calcium, magnesium, phosphate, and potassium in critically ill patients: a multicenter cohort study.” We have addressed all editorial and reviewer comments (numbered in italics), with responses in this letter (additions in blue), and have uploaded tracked-changes and clean versions of the manuscript, along with figures, tables, and supplement, to the journal submission website.

Editorial comments

1. Please ensure that your manuscript meets PLOS ONE's style requirements, including those for file naming.

We have adjusted formatting to ensure we meet the style requirements.

2. According to your manuscript, your waiver of ethical approval was secured from the Boston University Institutional Review Board (#H-41795). However, your wavier of ethical approval document has a different reference number (H-43331). Please provide the corrected reference number in your manuscript or provide an explanation for this discrepancy.

The correct number is H-43331 and the manuscript and attachment now match.

3. We note that you have indicated that there are restrictions to data sharing for this study. For studies involving human research participant data or other sensitive data, we encourage authors to share de-identified or anonymized data. However, when data cannot be publicly shared for ethical reasons, we allow authors to make their data sets available upon request. Before we proceed with your manuscript, please address the following prompts: a) If there are ethical or legal restrictions on sharing a de-identified data set, please explain them in detail (e.g., data contain potentially identifying or sensitive patient information, data are owned by a third-party organization, etc.) and who has imposed them (e.g., a Research Ethics Committee or Institutional Review Board, etc.). Please also provide contact information for a data access committee, ethics committee, or other institutional body to which data requests may be sent.

There are legal restrictions on sharing the data because they are owned by a third-party organization (Premier Inc), not the authors, and therefore the authors are not able to share the data. The database is accessible through Premier Inc (https://premierinc.com/real-world-evidence/real-world-data). Premier Inc charges for access to the database.

4. Please update your Data Availability statement in the submission form accordingly.

We have updated the Data Availability statement which now reads:

“The data use agreement between Premier Inc. and Boston University prevents sharing of the datasets used for this study. There are legal restrictions on sharing the data because they are owned by a third-party organization (Premier Inc), not the authors, and therefore the authors are not able to share the data. The database is accessible through Premier Inc (https://premierinc.com/real-world-evidence/real-world-data). Premier Inc charges for access to the database. Analytic code is available on the Open Science Framework (https://osf.io/ndjyh/overview).”

5. Please include captions for your Supporting Information files at the end of your manuscript, and update any in-text citations to match accordingly. Please see our Supporting Information guidelines for more information: http://journals.plos.org/plosone/s/supporting-information.

We have added a caption to “S1 Appendix”, updated in-text citations to match, and adjusted the file name.

Reviewer 1 comments:

Major comments.

6. The manuscript does not adequately address the underlying causes of abnormal electrolyte levels (e.g., sepsis, gastrointestinal losses, diuretic use, nutritional status, acid–base disorders). Without this context, it is difficult to interpret whether observed supplementation practices reflect appropriate responses to reversible conditions or empiric replacement. Can the authors discuss whether such factors were available in the database?

The reviewer is correct that our manuscript does not focus on the underlying causes of abnormal electrolyte levels. The Premier Healthcare Database does not have sufficient granularity about the time-sequence or nuance of the diagnoses that lead to electrolyte deficiency for these to be incorporated in the analysis. Even with that data, accepted clinical practice around electrolyte supplementation is often based on eminence rather than evidence, and so it would be difficult to know whether supplementation prompted by a given clinical scenario was beneficial or not. We have expanded our limitations section by adding the following:

“We did not attempt to correct for case-mix of critically ill patients or the underlying diagnoses, because the available data were insufficiently detailed to allow for clinically robust adjustment.”

7. Patients with acute or chronic renal failure were excluded, but the definitions are not sufficiently detailed.

If patients had a diagnosis of acute or chronic renal dysfunction based on their International Classification of Disease codes, then we excluded them. We have updated the Patients sub-section of the Methods section which now reads:

“We excluded patients with diagnoses of acute or chronic renal dysfunction (Section 2 in Appendix S1) because electrolyte supplementation may be less common in such patients; patients with a pregnancy-related diagnosis (ICD-10 O00-O9A) because of magnesium administration for pre-eclampsia(23); and patients with intracranial bleeding because some centers may administer magnesium to aid in cerebral hemostasis.”

And in Appendix S1 we now include a section reading: “If any of the following International Classification of Diseases (ICD) codes were present, then we classified a patient as having acute or chronic renal dysfunction: "N17.0", "N17.1", "N17.2", "N17.8", "N17.9", "I13x", "N18x", "N19x", "I12.0x", "N03.2x", "N03.3x", "N03.4x", "N03.5x", "N03.6x", "N03.7x", "N05.2x", "N05.3x", "N05.4x", "N05.5x", "N05.6x", "N05.7x", "N25.0x", "Z49.0x", "Z94.0x", "Z99.2x", "Z39.32x", "Z48.22x", "Z49.31x", "Z91.15x".”

8. The manuscript references mechanical ventilation in the discussion, yet the proportion of patients receiving mechanical ventilation in the cohort is not reported. Can the authors look at the frequency of mechanical ventilation?

We have added this information to Table 1. A total of 16,183 (33.6%) were receiving invasive mechanical ventilation on admission.

9. The cohort likely includes a heterogeneous mix of medical and surgical ICU patients. Please look at medical vs surgical admissions, and the number of patients undergoing major surgeries, such as cardiac or abdominal surgery

We have added a row showing the proportion of patients who had a major procedure or surgery on admission to Table 1 (15,992, 33.2%), and we have added information about surgical admissions to new supplemental table now numbered eTable 1 in S1 Appendix.

10. Can the authors please clarify whether admissions were elective or emergent?

The data include all patients admitted to the intensive care unit who had the studied electrolytes sampled. This includes both elective and emergent admissions.

11. Sodium is a commonly monitored and clinically important electrolyte in ICU patients. Can the authors justify why sodium was excluded from the analysis?

Sodium was not included because it is generally not supplemented based on the serum level, nor is it commonly an element of electrolyte supplementation protocols. We have added this explanation to the Methods section:

“We focused on levels and supplementation of calcium, magnesium, phosphate, and potassium, because these electrolytes are commonly measured in critically ill patients and commonly included in serum level-based supplementation protocols ordered at ICU admission. W We did not include other electrolytes such as sodium or zinc, which are uncommonly supplemented in a protocolized fashion.”

12. It is unclear why congestive heart failure (CHF) was not included among reported comorbidities, given its association with electrolyte abnormalities and supplementation decisions.

We have added congestive heart failure to the list of comorbidities. A total of 13,764 (28.7%) had congestive heart failure.

13. Related to this, how the term “cardiac arrhythmia” in table 1 was identified?

Cardiac arrhythmia was defined using ICD codes. If any of the following codes were present, we classified the patient as having cardiac arrhythmia. This was based on prior work now cited in the manuscript (10.1513/AnnalsATS.202111-1251RL, 10.1097/MLR.0000000000000824): "I47x", "I48x", "I49x", "I44.0x", "I44.1x", "I44.3x", "I44.4x", "I44.5x", "I44.6x", "I44.7x", "I44.8x", "I44.9x", "I45.0x", "I45.1x", "I45.2x", "I45.3x", "I45.4x", "I45.5x", "I45.6x", "I45.7x", "I45.8x", "I45.9x", "R00.0x", "R00.1x", "R00.8x", "T82.1x", "Z45.0x", "Z95.0x", "Z95.9x, "Z95.810x", "Z95.818x".

14. The manuscript lists “fluid and electrolyte disorders” as a comorbidity in table 1. Please clarify: 1) How this variable was defined (ICD codes?),

This variable included the following ICD codes: "E86x", "E87x", "E22.2x" and this is now explained in Section 2, S1 Appendix.

15. 2) Whether it overlaps with the electrolyte abnormalities measured in this study, and

Yes, we anticipate that there may have been overlap with electrolyte abnormalities seen on admission in patients included in this study.

16. 3) Whether inclusion of this variable risks circular reasoning or misclassification bias.

If our work had involved adjusting for baseline variables such as diagnosis or other clinical details, then there is a possibility that including redundant information (such as a diagnosis of fluid or electrolyte abnormalities) could introduce collider bias. We did not build any models to adjust for baseline characteristics, because our main goals were to describe frequency and its variation, within the limitations of unadjusted assessment.

Reviewer 2 comments:

17. The manuscript provides valuable descriptive data on electrolyte supplementation practices across U.S. ICUs, but its conceptual framing is limited by a narrow focus on serum values

We agree that serum levels of electrolytes do not necessarily correlate with total body stores, especially for electrolytes which are primarily found intracellularly or in tissues (e.g. calcium, magnesium, phosphate, potassium). We have updated the limitations section:

“Our study has important limitations. For the electrolytes studied, which are primarily intracellular or stored in tissue, serum levels are not necessarily correlated with total body stores.(5, 6, 49, 50) Calendar days…”

18. and it is not clear how supplementation was adminstered,

The top 3 supplementation regimens for each electrolyte are available in eTable 3 in the electronic supplement. We have made this more clear in the manuscript:

“The most common supplementation route was parenteral for calcium, magnesium, and phosphate, and oral for potassium (eTable 3 in S1 Appendix contains additional details about administered supplements).”

19. nor if it included dietician prescribed medical nutrition therapy.

We did not include electrolytes included in hospital food or enteral nutrition formulations ordered by the dietitian, and have added this to the limitations:

“We did not analyze calcium, magnesium, phosphate or potassium ingested as food or in enteral feeds, nor did we include this as supplementation.”

20. The study does not provide sufficient details of the role of physiologic mechanisms that drive electrolyte abnormalities in critical illness,

21. nor does it acknowledge that serum concentrations are poor proxies for tissue concentrations. For example, hypophosphatemia is common in hospitalized patients due to a shift of phosphate into tissue.

Please see response to comment 17.

22. Notably, the omission of sodium — the most commonly administered electrolyte and a major driver of iatrogenic harm from intravenous saline — further limits the comprehensiveness of the analysis. Indeed, hyponatremia is often misdiagnosed in hypervolemic conditions in which sodium concentrations are diluted by excessive fluid retention.

Please see response to comment 11.

23. The complex interaction of sodium levels with hypokalemia is also important.

Thank you for flagging this point. We do not feel it is possible to sufficiently address it within the conceptual and logistical space of this manuscript, but we have added the following to the future directions identified at the end:

“Future work should focus on observational and randomized studies aimed at distinguishing between beneficial and unnecessary or harmful supplementation, with careful attention to clinical factors that may generate heterogeneity, to improve care for all critically ill patients.”

24. Furthermore, the paper treats supplementation as benign and does not sufficiently focus on their potential adverse effects associated with aggressive dosages.

We agree that supplementation is not necessarily benign, and part of our overall goal in performing this work was to call into question the benefit of ubiquitous supplementation. We have revised the start of the discussion section which now reads: “The ubiquity and variability of supplementation highlight an opportunity to improve care for critically ill patients by increasing beneficial supplementation and reducing unnecessary or harmful supplementation.”, and the last sentence of the manuscript (see response to comment 23).

25. Overall, the paper would benefit from a more physiologically grounded discussion of the well-documented pathophysiological causes related to electrolyte dysregulation and a clearer acknowledgment that supplementation practices only superficially target serum numbers rather than underlying pathophysiology. Such a grounded discussion could eventually contribute to correcting the identified inconsistencies in supplementation practices in ICUs.

26. Finally, the authors call for randomized controlled trials, but such clinical designs make little sense without a more comprehensive understanding of the underlying causative factors that disturb electrolyte homeostasis in critically ill patients.

Our perspective in calling for randomized trials is that this is the study design that will minimize bias from confounding by indication and will generate the strength of evidence needed to change practice. We have left in the call for randomization but added observational studies as well: “Future work should focus on observational and randomized studies aimed at distinguishing between beneficial and unnecessary or harmful supplementation, with careful attention to clinical factors that may generate heterogeneity, to improve care for all critically ill patients.”

Thank you for the opportunity to revise and resubmit.

Sincerely,

Christopher Yarnell MD PhD

Attachment Submitted filename: CaMgKPhos Plos One Response to Reviewers R1.docx

# Peer review: Decision Letter 1

**DOI:** 10.1371/journal.pone.0349145.r003

**Authors:** Mohammed S. Razzaque

8 Apr 2026

PONE-D-26-00828R1

Supplementation of calcium, magnesium, phosphate, and potassium in critically ill patients: a multicenter cohort study

PLOS One

Dear Dr. Yarnell,

Thank you for submitting your manuscript to PLOS ONE. After careful consideration, we feel that it has merit but does not fully meet PLOS ONE’s publication criteria as it currently stands. Therefore, we invite you to submit a revised version of the manuscript that addresses the points raised during the review process.

Please submit your revised manuscript by May 23 2026 11:59PM. If you will need more time than this to complete your revisions, please reply to this message or contact the journal office at plosone@plos.org. When you're ready to submit your revision, log on to https://www.editorialmanager.com/pone/ and select the 'Submissions Needing Revision' folder to locate your manuscript file.

Please include the following items when submitting your revised manuscript:

- A letter that responds to each point raised by the academic editor and reviewer(s). You should upload this letter as a separate file labeled 'Response to Reviewers'.
- A marked-up copy of your manuscript that highlights changes made to the original version. You should upload this as a separate file labeled 'Revised Manuscript with Track Changes'.
- An unmarked version of your revised paper without tracked changes. You should upload this as a separate file labeled 'Manuscript'.

If you would like to make changes to your financial disclosure, please include your updated statement in your cover letter. Guidelines for resubmitting your figure files are available below the reviewer comments at the end of this letter.

If applicable, we recommend that you deposit your laboratory protocols in protocols.io to enhance the reproducibility of your results. Protocols.io assigns your protocol its own identifier (DOI) so that it can be cited independently in the future. For instructions see: https://journals.plos.org/plosone/s/submission-guidelines#loc-laboratory-protocols. Additionally, PLOS ONE offers an option for publishing peer-reviewed Lab Protocol articles, which describe protocols hosted on protocols.io. Read more information on sharing protocols at https://plos.org/protocols?utm_medium=editorial-email&utm_source=authorletters&utm_campaign=protocols.

As the corresponding author, your ORCID iD is verified in the submission system and will appear in the published article. PLOS supports the use of ORCID, and we encourage all coauthors to register for an ORCID iD and use it as well. Please encourage your coauthors to verify their ORCID iD within the submission system before final acceptance, as unverified ORCID iDs will not appear in the published article. *Only*  the individual author can complete the verification step; PLOS staff *cannot*  verify ORCID iDs on behalf of authors.

We look forward to receiving your revised manuscript.

Kind regards,

Mohammed S. Razzaque, MBBS, PhD

Academic Editor

PLOS One

Journal Requirements:

If the reviewer comments include a recommendation to cite specific previously published works, please review and evaluate these publications to determine whether they are relevant and should be cited. There is no requirement to cite these works unless the editor has indicated otherwise.

Please review your reference list to ensure that it is complete and correct. If you have cited papers that have been retracted, please include the rationale for doing so in the manuscript text, or remove these references and replace them with relevant current references. Any changes to the reference list should be mentioned in the rebuttal letter that accompanies your revised manuscript. If you need to cite a retracted article, indicate the article’s retracted status in the References list and also include a citation and full reference for the retraction notice.

Additional Editor Comments (if provided):

Please explain why sodium assessment was not included in the study and highlight it as a potential limitation.

[Note: HTML markup is below. Please do not edit.]

Reviewers' comments:

Reviewer's Responses to Questions

**Comments to the Author**

1. If the authors have adequately addressed your comments raised in a previous round of review and you feel that this manuscript is now acceptable for publication, you may indicate that here to bypass the “Comments to the Author” section, enter your conflict of interest statement in the “Confidential to Editor” section, and submit your "Accept" recommendation.

Reviewer #1: (No Response)

Reviewer #2: (No Response)

**********

2. Is the manuscript technically sound, and do the data support the conclusions?

The manuscript must describe a technically sound piece of scientific research with data that supports the conclusions. Experiments must have been conducted rigorously, with appropriate controls, replication, and sample sizes. The conclusions must be drawn appropriately based on the data presented.

Reviewer #1: Yes

Reviewer #2: Yes

**********

3. Has the statistical analysis been performed appropriately and rigorously?

Reviewer #1: Yes

Reviewer #2: Yes

**********

4. Have the authors made all data underlying the findings in their manuscript fully available?

The PLOS Data policy requires authors to make all data underlying the findings described in their manuscript fully available without restriction, with rare exception (please refer to the Data Availability Statement in the manuscript PDF file). The data should be provided as part of the manuscript or its supporting information, or deposited to a public repository. For example, in addition to summary statistics, the data points behind means, medians and variance measures should be available. If there are restrictions on publicly sharing data—e.g. participant privacy or use of data from a third party—those must be specified.

Reviewer #1: Yes

Reviewer #2: Yes

**********

5. Is the manuscript presented in an intelligible fashion and written in standard English?

PLOS ONE does not copyedit accepted manuscripts, so the language in submitted articles must be clear, correct, and unambiguous. Any typographical or grammatical errors should be corrected at revision, so please note any specific errors here.

Reviewer #1: Yes

Reviewer #2: Yes

**********

6. Review Comments to the Author

Please use the space provided to explain your answers to the questions above. You may also include additional comments for the author, including concerns about dual publication, research ethics, or publication ethics. (Please upload your review as an attachment if it exceeds 20,000 characters)

Reviewer #1: I appreciate the authors’ explanation for excluding sodium; however, I remain concerned that this omission limits the completeness of a descriptive study of electrolyte management in the ICU. Hyponatremia is common and clinically important in critically ill patients, and its correction—particularly in moderate to severe cases—reflects active management decisions and likely inter‑hospital variation, even if not protocolized in the same manner as other electrolytes. Inclusion of sodium would enhance the clinical relevance of the study.

Reviewer #2: Regarding sodium in Comment 11:

The authors' rationale for excluding sodium requires correction. The manuscript states:

“Sodium was not included because it is generally not supplemented based on the serum level…”

This is inaccurate. In ICU practice, serum sodium is routinely monitored and sodium is supplemented accordingly—most commonly through intravenous fluids such as normal saline. Thus, the current wording implies a misunderstanding of standard electrolyte management.

Please revise to clarify that sodium was excluded not because it is rarely supplemented, but because its supplementation is embedded within fluid management rather than ordered as a discrete electrolyte replacement, and therefore falls outside the scope of the present analysis of discretionary supplementation.

Regarding Comment 23:

The response does not fully address the broader point that electrolyte supplementation in ICU patients is influenced by interactions with other supplements, dietary factors, and concurrent medications. These interactions can generate clinical harm or interfere with medical treatments. The manuscript should note that future studies should examine these supplement interactions, as they represent a potential source of adverse effects in electrolyte management.

Regarding Comment 25:

The authors’ made no response to acknowledge a central limitation of electrolyte supplementation which often treats superficial serum fluctuations without addressing the underlying homeostatic disturbances that generate them. A brief acknowledgment of this limitation is needed along with a call for further investigations of pathophysiology mechanisms associated with electrolyte dysregulation. Clinical trials and observational studies alone cannot address the need for basic research in this area.

**********

7. PLOS authors have the option to publish the peer review history of their article (what does this mean?). If published, this will include your full peer review and any attached files.

If you choose “no”, your identity will remain anonymous but your review may still be made public.

**Do you want your identity to be public for this peer review?**  For information about this choice, including consent withdrawal, please see our Privacy Policy.

Reviewer #1: No

Reviewer #2: No

**********

[NOTE: If reviewer comments were submitted as an attachment file, they will be attached to this email and accessible via the submission site. Please log into your account, locate the manuscript record, and check for the action link "View Attachments". If this link does not appear, there are no attachment files.]

To ensure your figures meet our technical requirements, please review our figure guidelines: https://journals.plos.org/plosone/s/figures

You may also use PLOS’s free figure tool, NAAS, to help you prepare publication quality figures: https://journals.plos.org/plosone/s/figures#loc-tools-for-figure-preparation.

NAAS will assess whether your figures meet our technical requirements by comparing each figure against our figure specifications.

# Author comment: Author response to Decision Letter 2

**DOI:** 10.1371/journal.pone.0349145.r004

15 Apr 2026

Please see attached response to reviewers letter for a potentially more readable version of the responses below.

Dear Editor Dr Mohammed S. Razzaque and the PLOS One Editorial Team,

Thank you for the opportunity to revise our manuscript, “Supplementation of calcium, magnesium, phosphate, and potassium in critically ill patients: a multicenter cohort study.” We have addressed all editorial and reviewer comments (numbered in italics), with responses in this letter (additions in blue), and have uploaded tracked-changes and clean versions of the manuscript, along with figures, tables, and supplement, to the journal submission website.

Editorial comments:

1. Please explain why sodium assessment was not included in the study and highlight it as a potential limitation.

We did not include sodium assessment in the study because sodium is not an electrolyte that is commonly treated with discrete supplementation in a protocolized fashion according to serum levels in critically ill patients. Hyponatremia treatment sometimes involves sodium supplementation, when the etiology of the hyponatremia is inappropriate antidiuretic hormone activity and fluid restriction alone does not suffice. Description of the epidemiology of sodium and its supplementation would be a worthwhile project for a separate manuscript. Other electrolytes that we did not discuss in this work include chloride, bicarbonate, and zinc.

We adjusted the following sentence in the Methods:

“We did not include other electrolytes such as sodium, chloride, bicarbonate, or zinc, which are uncommonly supplemented in a protocolized fashion.”

We added the following limitation to the discussion:

“We did not include all electrolytes relevant for critical illness, but instead focused on those commonly treated with discrete supplements administered in a protocolized fashion. Notable omissions include sodium, bicarbonate, chloride, and zinc.”

Reviewer 1 comments:

2. Reviewer #1: I appreciate the authors’ explanation for excluding sodium; however, I remain concerned that this omission limits the completeness of a descriptive study of electrolyte management in the ICU. Hyponatremia is common and clinically important in critically ill patients, and its correction—particularly in moderate to severe cases—reflects active management decisions and likely inter‑hospital variation, even if not protocolized in the same manner as other electrolytes. Inclusion of sodium would enhance the clinical relevance of the study.

Please see response to editor comment 1. Description of the epidemiology of sodium and its supplementation, including investigation of inter-hospital variation, would be a worthwhile project for a separate manuscript. Sodium supplementation intended to expand the intravascular volume is different from protocolized supplementation of calcium, magnesium, phosphate, or potassium based on level. Sodium supplementation for hyponatremia has separate nuances around rate of correction and extravascular fluid status (hyper- or hypovolemic), which are distinct from the nuances of the studied electrolytes.

Reviewer 2 comments:

3. Reviewer #2: Regarding sodium in Comment 11: The authors' rationale for excluding sodium requires correction. The manuscript states: “Sodium was not included because it is generally not supplemented based on the serum level…” This is inaccurate. In ICU practice, serum sodium is routinely monitored and sodium is supplemented accordingly—most commonly through intravenous fluids such as normal saline. Thus, the current wording implies a misunderstanding of standard electrolyte management. Please revise to clarify that sodium was excluded not because it is rarely supplemented, but because its supplementation is embedded within fluid management rather than ordered as a discrete electrolyte replacement, and therefore falls outside the scope of the present analysis of discretionary supplementation.

Thank you for this point. We agree that sodium supplementation is common but distinct from the supplementation used for the studied electrolytes. Please see response to Editor comment 1 for further details and our addition to the Limitations section.

4. Regarding Comment 23: The response does not fully address the broader point that electrolyte supplementation in ICU patients is influenced by interactions with other supplements, dietary factors, and concurrent medications. These interactions can generate clinical harm or interfere with medical treatments. The manuscript should note that future studies should examine these supplement interactions, as they represent a potential source of adverse effects in electrolyte management.

We have adjusted the final paragraph of the discussion (just prior to the conclusion), which now reads:

“Given the heterogeneity of electrolyte supplementation practices, and the complexity of potential interactions between concurrent medications, dietary factors, and other supplements, several types of studies are needed to help improve electrolyte management for critically ill patients. Physiological and observational studies can help clarify the nature of interactions and mechanisms for both benefit and harm with supplementation. Randomized controlled trials can compare…”

5. Regarding Comment 25: The authors’ made no response to acknowledge a central limitation of electrolyte supplementation which often treats superficial serum fluctuations without addressing the underlying homeostatic disturbances that generate them. A brief acknowledgment of this limitation is needed along with a call for further investigations of pathophysiology mechanisms associated with electrolyte dysregulation. Clinical trials and observational studies alone cannot address the need for basic research in this area.

We agree that serum fluctuations do not necessarily correlate with underlying deficiency and therefore the entire enterprise of level-based electrolyte supplementation may be flawed. We have added the following to the Limitations paragraph:

“We did not analyze the association between deficiencies or supplementation and outcomes such as duration of ventilation or mortality, because it was not possible to account for enough of the relevant confounding variables with the available data. Consequently, our study does not shed light on a central limitation of electrolyte supplementation based on serum levels, which is that variation in serum levels may be only loosely correlated with total body stores, and therefore supplementation based on level might not change clinical outcomes.”

We also made an addition to the final Conclusions paragraph:

“Future work should focus on physiological, observational, and randomized studies aimed at distinguishing between beneficial and unnecessary or harmful supplementation, with careful attention to clinical factors that may generate heterogeneity, to improve care for all critically ill patients.”

We also mentioned physiological studies in the future directions paragraph, as outlined in response to reviewer comment 4 above.

Thank you for the opportunity to revise and resubmit.

Sincerely,

Christopher Yarnell MD PhD

Attachment Submitted filename: CaMgKPhos Plos One Response to Reviewers R2.docx

# Editor report: Decision Letter 2

**DOI:** 10.1371/journal.pone.0349145.r005

**Authors:** Mohammed S. Razzaque

26 Apr 2026

Supplementation of calcium, magnesium, phosphate, and potassium in critically ill patients: a multicenter cohort study

PONE-D-26-00828R2

Dear Dr. Yarnell,

We’re pleased to inform you that your manuscript has been judged scientifically suitable for publication and will be formally accepted for publication once it meets all outstanding technical requirements.

Within one week, you’ll receive an e-mail detailing the required amendments. When these have been addressed, you’ll receive a formal acceptance letter and your manuscript will be scheduled for publication.

An invoice will be generated when your article is formally accepted. Please note, if your institution has a publishing partnership with PLOS and your article meets the relevant criteria, all or part of your publication costs will be covered. Please make sure your user information is up-to-date by logging into Editorial Manager at Editorial Manager® and clicking the ‘Update My Information' link at the top of the page. For questions related to billing, please contact billing support.

If your institution or institutions have a press office, please notify them about your upcoming paper to help maximize its impact. If they’ll be preparing press materials, please inform our press team as soon as possible -- no later than 48 hours after receiving the formal acceptance. Your manuscript will remain under strict press embargo until 2 pm Eastern Time on the date of publication. For more information, please contact onepress@plos.org.

Kind regards,

Mohammed S. Razzaque, MBBS, PhD

Academic Editor

PLOS One

Additional Editor Comments (optional):

Reviewers' comments:

# Editor report: Acceptance letter

**DOI:** 10.1371/journal.pone.0349145.r006

**Authors:** Mohammed S. Razzaque

PONE-D-26-00828R2

PLOS One

Dear Dr. Yarnell,

I'm pleased to inform you that your manuscript has been deemed suitable for publication in PLOS One. Congratulations! Your manuscript is now being handed over to our production team.

At this stage, our production department will prepare your paper for publication. This includes ensuring the following:

* All references, tables, and figures are properly cited

* All relevant supporting information is included in the manuscript submission,

* There are no issues that prevent the paper from being properly typeset

You will receive further instructions from the production team, including instructions on how to review your proof when it is ready. Please keep in mind that we are working through a large volume of accepted articles, so please give us a few days to review your paper and let you know the next and final steps.

Lastly, if your institution or institutions have a press office, please let them know about your upcoming paper now to help maximize its impact. If they'll be preparing press materials, please inform our press team within the next 48 hours. Your manuscript will remain under strict press embargo until 2 pm Eastern Time on the date of publication. For more information, please contact onepress@plos.org.

You will receive an invoice from PLOS for your publication fee after your manuscript has reached the completed accept phase. If you receive an email requesting payment before acceptance or for any other service, this may be a phishing scheme. Learn how to identify phishing emails and protect your accounts at https://explore.plos.org/phishing.

If we can help with anything else, please email us at customercare@plos.org.

Thank you for submitting your work to PLOS ONE and supporting open access.

Kind regards,

PLOS ONE Editorial Office Staff

on behalf of

Dr. Mohammed S. Razzaque

Academic Editor

PLOS One
